# Deciphering molecular determinants of GPBAR1-Gs protein interactions by HDX-MS and cryo-EM

**DOI:** 10.1038/s41598-025-16529-w

**Published:** 2025-08-26

**Authors:** Jérôme Castel, Thomas Botzanowski, Ieva Brooks, Alexandre Frechard, Gilbert Bey, Marine Schroeter, Elise Del Nero, François Debaene, Fabrice Ciesielski, Denis Zeyer, Sarah Cianferani, Renaud Morales

**Affiliations:** 1 Department of Biophysics, Novalix, 16 Rue d’Ankara, 67000 Strasbourg, France; 2 Laboratoire de Spectrométrie de Masse BioOrganique, IPHC UMR 7178, Infrastructure Nationale de Protéomique ProFI, Université de Strasbourg, CNRS, UAR2048 CNRS, 67087 Strasbourg, France; 3 Department of Structural Biology, Novalix, 16 Rue d’Ankara, 67000 Strasbourg, France

**Keywords:** GPBAR1, GPCR, G protein, GPCR-G protein interactions, HDX-MS, cryo-EM, Mass spectrometry, Cryoelectron microscopy, Mass spectrometry, G protein-coupled receptors

## Abstract

Many physiological processes are dependent on G protein-coupled receptors (GPCRs), the biggest family of human membrane proteins and a significant class of therapeutic targets. Once activated by external stimuli, GPCRs use G proteins and arrestins as transducers to generate second messengers and trigger downstream signaling, leading to diverse signaling profiles. The G protein-coupled bile acid receptor 1 (GPBAR1, also known as Takeda G protein-coupled receptor 5, TGR5) is a class A bile acid membrane receptor that regulates energy homeostasis and glucose and lipid metabolism. GPBAR1/Gs protein interactions are implicated in the prevention of diabetes and the reduction of inflammatory responses, making GPBAR1 a potential therapeutic target for metabolic disorders. Here, we present combined hydrogen/deuterium exchange mass spectrometry (HDX-MS) and cryo-electron microscopy (cryo-EM) to identify the molecular determinants of GPBAR1 conformational dynamics upon G protein binding. Thanks to extensive optimization, we achieved over 75% sequence coverage by HDX-MS of a complete GPCR complex and a 2.5 Å resolution structure by cryo-EM, both of which are state-of-the-art. Altogether, our results provide information on the under-investigated GPBAR1 binding mode to its cognate G protein, pinpointing the synergic and powerful combination of higher cryo-EM and lower HDX-MS resolution techniques to dissect GPCR/G protein binding characteristics.

**Supplementary Information:**

The online version contains supplementary material available at 10.1038/s41598-025-16529-w.

## Introduction

G protein-coupled receptors (GPCRs) constitute the largest and the most diverse family of cell-surface receptors, with around 800 identified in humans. They play an essential role in the regulation of physiological functions (such as vision, olfactory perception, neurotransmission, pain, immunity…). Their dysfunctions or mutations are associated with a variety of diseases (Alzheimer’s disease, hypertension, bronchial asthma, multiple sclerosis, diabetes, and so on). GPCRs are, therefore, widely recognized as important therapeutic targets for pharmaceutical development, with 30–40% of all drugs approved by the US Food and Drug Administration (FDA) known to specifically target GPCRs. From a structural point of view, all GPCRs share a common architecture composed of seven transmembrane α-helices (TM1-TM7) connected by three extracellular loops (ECLs) and three intracellular loops (ICLs). GPCRs have an extracellular N-terminus that can participate in ligand binding, especially in classes B and C, while the intracellular C-terminus interacts with various intracellular signaling proteins, including G proteins, kinases, and arrestins.

GPCR activation is induced by extracellular signals, such as photons, ligands, or hormones, allowing them to interact with signaling molecules^1–3^, among which the G proteins are the best characterized^4^. These are heterotrimeric complexes composed of alpha (Gα), beta (Gβ), and gamma (Gγ) subunits. G proteins can be distinguished by their Gα subunits, which are grouped into four families based on sequence similarities and functional results (Gαs, Gαi/o, Gαq/11, and Gα12/13)^5^. In the basal (resting) state, the Gα subunit, whose catalytic site is occupied by a guanosine diphosphate (GDP) nucleotide, interacts with the Gβ and Gγ subunits. Activation of G proteins and initiation of signaling cascades are achieved by nucleotide dissociation from the Gα subunit. The interaction of a GPCR with a G protein triggers the exchange of GDP into guanosine triphosphate (GTP), followed by dissociation of the Gα subunit first and secondly the Gβ/Gγ subunits. The GTP-bound Gα subunit or Gβ/γ subunits then interact with and regulate effector proteins (ion channels, phospholipases.)^6,7^. Although some GPCRs interact with a single G protein, many can bind to one or more Gα subunits, inducing the activation of several effector proteins^8,9^.

The structural characterization of GPCR/G protein (GPCR/G) interactions enables understanding of GPCR signaling mechanisms and the development of new drugs targeting these receptors. X-ray crystallography, cryo-EM^10,11^, and NMR have been the predominant methodologies deployed to obtain high-resolution 3D structures and the study of GPCRs complexed to ligands and G proteins. The first structure of a GPCR/G protein complex was obtained by crystallography in 2011, revealing β2A adrenergic receptor interacting with a heterotrimeric Gs protein in a nucleotide-free state and stabilized by a nanobody (Nb35)^12^. Given the challenges in stabilizing GPCR/G complexes in an active conformation, several strategies have been developed. First, apyrase was used to trap the GPCR/G complex in a nucleotide-free state^11,13^. Secondly, a single-domain antibody (nanobody) was added to stabilize the interface between the Gβ/Gγ and Gɑ subunits within the G protein, thus stabilizing the GPCR/G complex^14,15^. Subsequently, technological breakthroughs in cryo-EM have enabled the resolution of numerous GPCR/G structures with the Gs subfamily (CTR^16^, GLP1^17^, etc.), Gi (opsin^18^, MO^19^, endothelin-1-ETB^20^ or Go (5HT1BR^21^. However, most of these structures are static snapshots of the most stable state and do not provide complete visualization of conformational states and the dynamics of GPCRs. Indeed, most of the transient and metastable states present in solution are often not captured in crystallography or cryo-EM. Over the past two decades, MS has established itself as a powerful complementary approach to the more conventional high-resolution biophysical techniques used in structural biology to probe the structural architecture and dynamics of GPCRs and their related complexes^22–24^. In particular, MS has been used to understand the ability of different molecules or ligands to modulate the structural dynamics of GPCRs, including during the formation of complexes between G proteins and receptors^25–27^. HDX-MS provides valuable and essential information on the dynamics and mechanisms leading to complex formation between GPCRs and GPCR/G complexes^28–30^. HDX-MS monitors solvent exchange by exposing the hydrogens within a protein molecule to a solvent containing the heavy hydrogen isotope – deuterium. This approach is a powerful tool for studying the stability, folding, conformational dynamics, and binding regions of proteins. Since the first use of HDX-MS on a GPCR/G complex involving β2A^31^ in 2011, additional HDX-MS studies have been carried out on several GPCRs. These have led to a better understanding of the structural mechanism linking complex formation and GDP nucleotide release^31^, as well as highlighting the existence of transient interactions during the coupling process^32,33^.

Here, we used full-length active GPBAR1 GPCR bound to its agonist P395 ligand in interaction with the full heterotrimeric G protein complex (comprising Gαs, Gβ, and Gγ subunits; and stabilized by Nb35) illustrating the synergy and complementarity of HDX-MS and cryo-EM integration in revealing the molecular determinants and interaction dynamics of GPCR/G protein. GPBAR1 is a class A bile acid membrane receptor that regulates energy homeostasis and glucose and lipid metabolism. In particular, GPBAR1/Gs interactions are involved in the prevention of diabetes and the reduction of inflammatory responses, making GPBAR1 a potential therapeutic target for several diseases, such as obesity or atherosclerosis. We present a high-resolution 2.5 Å cryo-EM structure of the GPBAR1/G complex, along with a comprehensive HDX-MS derived overview of the conformational dynamics while interacting with its Gs subunits.

## Results

### Purification and intact mass analysis of the GPBAR1/Gs complex

**Fig. 1:**
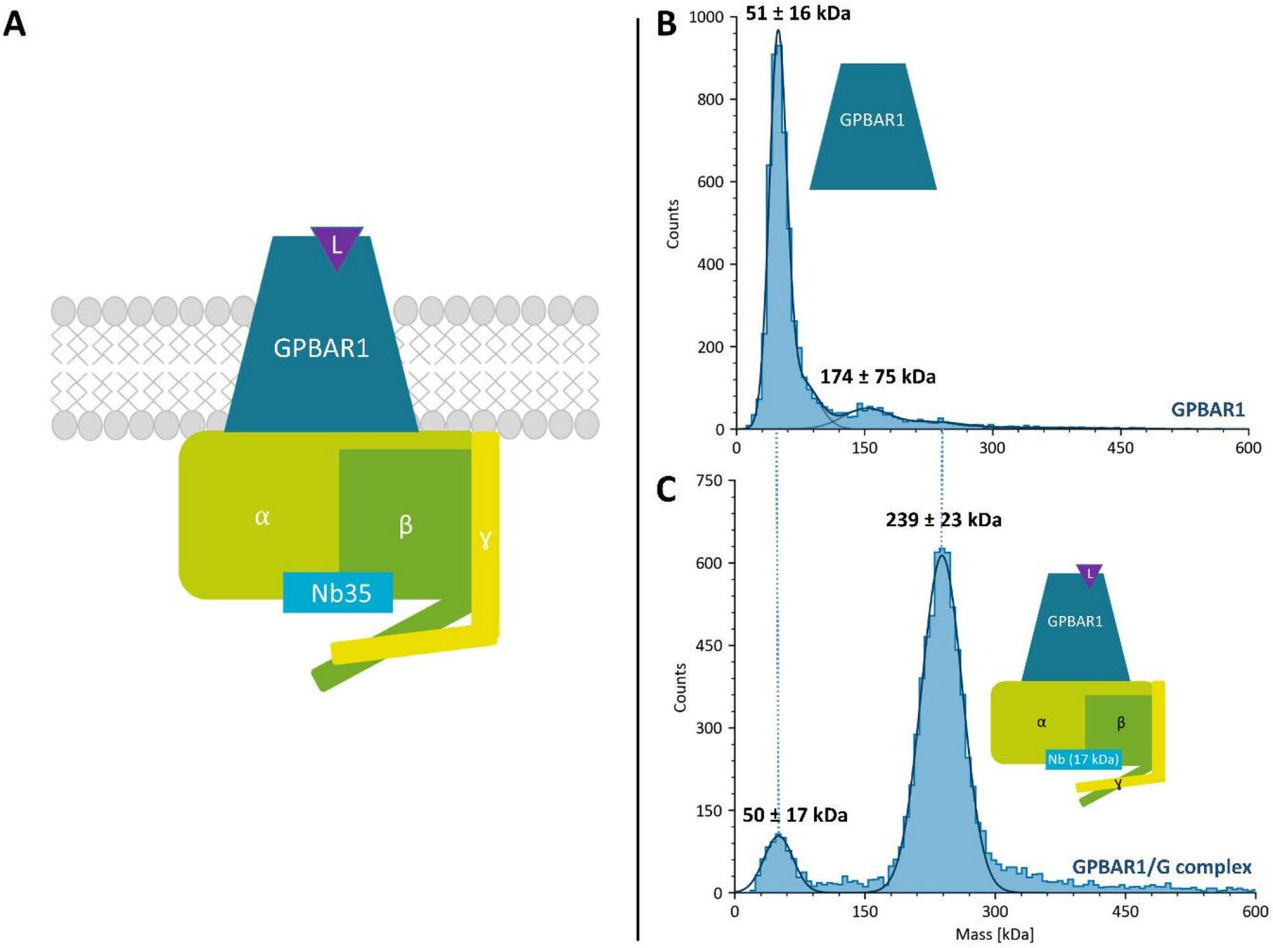
MPhoto analyses of GPBAR1 alone and the GPBAR1/Gs complex. (A) Schematic representation of the GPBAR1/Gs complex. The different G protein subunits are shown: Gαs in light green (43 kDa), Gβ in dark green (39 kDa), and Gγ (8 kDa) in yellow. The complex is stabilized by Nb35 (17 kDa), shown in cyan. (B). Histogram of the mass distribution of the GPBAR1 for a final concentration at 10 nM. (C) Histogram of the mass distribution of the GPBAR1/Gs complex for a final concentration at 30 nM. The continuous curve corresponds to the mass distributions of the majority of species fitted with a Gaussian function.

To form the active GPBAR1/Gs complex, GPBAR1 was co-expressed with Gαs_s_, Gβ, and Gγ in Sf21 insect cells and purified in the presence of P395, a highly potent synthetic agonist (see Materials and Methods for details). The complex was further stabilized using the camelid antibody, Nb35, which binds at the Gαs-Gβ interface. To assess the purity and homogeneity of the different protein assemblies, we first performed a 1D SDS-PAGE that revealed homogeneous Gs-heterotrimer and GPBAR1/Gs complex, demonstrating the purity of the samples (Supplementary data, Figure S1). To assess the GPBAR1/Gs complex integrity, an intact mass analysis using native mass photometry (MPhoto) was performed. MPhoto is a label-free single-molecule level technique particularly suited to the assessment of membrane protein mass in native environments (Fig. 1, and Supplementary data, Figure S2)^34,35^. For GPBAR1, MPhoto shows one single peak at 51 ± 16 kDa, in agreement with the theoretical mass (50.8 kDa) (Fig. 1.B). A minor species at 174 ± 75 kDa is also detected at higher mass, which could be attributed to GPBAR1 aggregation during sample dilution since the detergent concentration is below the critical micelle concentration (CMC). For the GPBAR1/G complex, MPhoto reveals a single sharp peak with a mass of 239 ± 23 kDa (theoretical mass ~ 158 kDa), suggesting a 1:1 GPBAR1/Gs complex included in LMNG micelles (Fig. 1.C). Indeed, mass differences of 50–80 kDa have already been reported for LMNG micelles (50–80 kDa)^36^. Although it cannot be ruled out that this single peak is composed of a mixture of empty and GPBAR1/G_s_-filled LMNG micelles, MPhoto, supported by 1D SDS-PAGE, is consistent with the formation of a 1:1 GPBAR1:G_s_ complex. Note that a minor (∼10%) distribution at 50 ± 17 kDa is also detected, which could correspond to an excess of G protein subunits present in the solution or a slight dissociation upon dilution.

### Cryo-EM structure of the GPBAR1/Gs complex

**Fig. 2:**
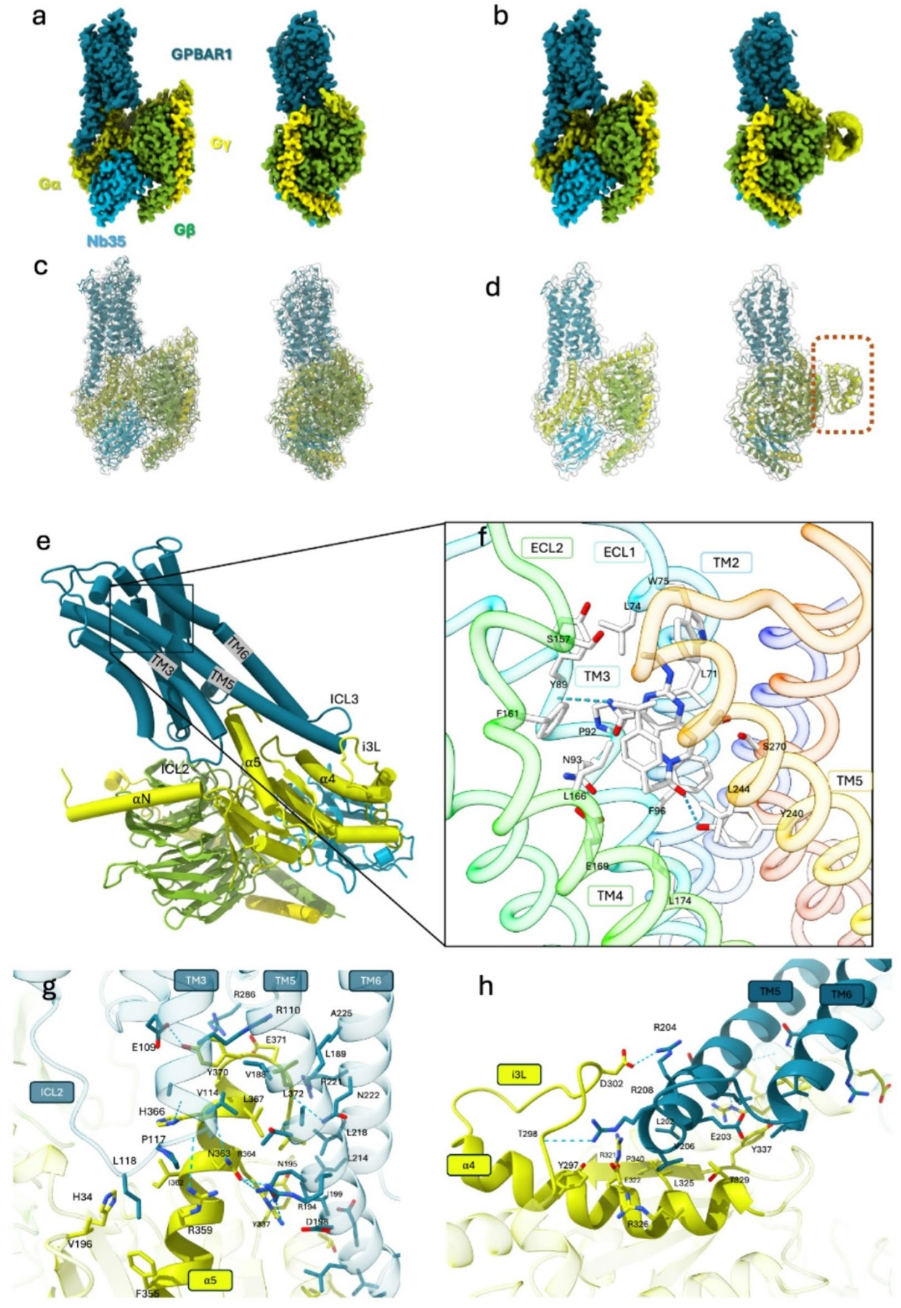
Cryo-EM map and atomic model. (a) Cryo-EM map at 2.5 Å of the GPBAR1 G protein complex, the map has been colored according to the different sub-units. (b) Cryo-EM map at 2.9 Å with the flexible AH domain. (c, d) Atomic model of the two complexes placed in the density. (d) The flexible Gαs part is surrounded by dotted lines. (e) Important regions for the interaction between GPBAR1 and Gαs. (f) P395 and the interacting residues of the ligand pocket, the backbones is rainbow colored (Blue N-ter Red C-ter). (g) Contact residues between GPBAR1 and the Gαs α5 helix. (h) Contact residues between TM5 and ICL3 of GPBAR1 with α4 and i3L of Gαs.

Cryo-EM gave high-resolution 3D atomic structures of the GPBAR1/Gs complex bound to the P395 ligand and the Nb35 nanobody. The first one, referred to as the “core structure” achieved the highest average resolution of 2.5 Å (Fig. 2.a, c), showing all the stable parts of the structure, including the P395 ligand and the nanobody. The second structure, with an average resolution of 2.9 Å (Fig. 2.b, d), is very similar to the core structure but additionally reveals the flexible, weakly resolved α-helical domain (Fig. 2.d) and will be further referred to as the “flex domain structure.”

Because of its higher resolution, the core structure was further used to investigate the interactions within the GPBAR1/Gs complex. GPBAR1 consists of seven transmembrane domains (TM), three cytosolic loops (ICL), and three extracellular loops. TM3, TM5, and TM6 surround α5 helix of Gαs (Fig. 2.g), while TM5, TM6, and ICL3 interact with α4 and i3L of Gαs (Fig. 2.h), incorporating GPBAR1 into the complex. The high resolution of the structure gives precise positioning of the ligand. The ligand pocket is formed by 16 residues: L71, L74, W75, Y89, P92, N93, F96, S157, F161, L166, E169, Y240, L244, S247, and S270. Most of the contacts are hydrophobic, but a hydrogen bond is formed between the nitrogen of the ligand’s carboxyl amine group and the main chain carboxyl group of Y89. Additionally, the hydroxyl group of Y240 forms a hydrogen bond with the ketone group of P395 ligand (Fig. 2.e, f).

GPBAR1 incorporates into the complex by interacting with the last two helices of Gαs, α5-α4, and the loop i3L (Fig. 2.g, h). The GPBAR1 residues involved in the interaction with α5 helix of Gαs are R110, E109, A113, and V114 from TM3; L118 and P117 from ICL2; V188, L189, T191, R194, N195, I199, and D198 from TM5; and L214, L218, R221, N222, and A225 from TM6. The remaining GPBAR1 contacts are made by the beginning of TM5 and ICL3, where arginine 204 and 208 form hydrogen bonds with D302 and T298 of Gαs i3L. Additionally, residues L202, E203, and V206 contact R321, D322, L325, R326, and T329 of α4 helix and Y337 of β6 of Gαs.

The α-helical (AH) domain (residues 67 to 171) of the “flex domain structure” shows the only major difference compared to the “core structure.” The low resolution of this domain (Supplementary data, Figure S3) and the fact that it does not appear in all the classes of the 3D classification step (Supplementary data, Figure S4) reflects its high flexibility. This is in line with previous studies showing that the AH domain delocalizes from the GαsRas domain and becomes flexible in the absence of nucleotides^37^. This flexibility is considered to be functionally important, allowing the AH domain to undergo essential conformational adjustments for efficient signaling. Note that for the rest of the “flex domain structure,” the organization and contacts between the different sub-units are the same as before.

### HDX-MS for conformational dynamics of GPBAR1/Gs complex

To probe the dynamics of GPBAR1/Gs formation, differential ΔHDX-MS data were generated.

#### Optimization of HDX-MS and consistency of the dataset

As sequence coverage (SC) and redundancy (R) are of utmost importance upstream from HDX-MS experiments, we first carried out an exhaustive optimization of the pre-analytical steps of the HDX-MS workflow on apo GPBAR1 in LMNG, including the amount injected, the nature of the protease and the quenching buffer to achieve the optimum conditions for concomitantly yielding the highest number of peptides, best sequence coverage, and highest redundancy (Supplementary data, Figures S5 and S6). HDX-MS optimization (final experimental conditions in supplementary data, Table S1) led to the identification of 332 peptides covering > 98% of the GPBAR1 sequence, with an average peptide redundancy of 8.8 (Supplementary data, Figure S7), which is quite remarkable and state-of-the-art for GPCRs. These numbers dropped slightly in the context of the final differential HDX (ΔHDX) experiments comparing apo GPBAR1 with ligand-bound GPBAR1/Gs complex, with 117 (out of 160) peptides retained after manual curation, representing a final sequence coverage of 76.4% and a redundancy of 3.8 (Supplementary data, Table S2).

To evaluate the consistency of the HDX-MS experiment, the interaction between the Nb35 nanobody and G protein subunits (Gαs and Gβ) was checked. Indeed, the paratope regions of the nanobody were well recognized by the G protein (Supplementary data, Figure S8). As a second internal control, peptides of the thermostabilized apocytochrome b562RIL (BRIL) sequence of GPBAR1 (a tag that helps for GPBAR1 production and purification) showed no difference in D incorporation between the GPBAR1/Gs and GPBAR1 alone (see supplementary data, Figure S9).

#### Differential HDX-MS (ΔHDX) of Apo GPBAR1 and GPBAR1/G complex support GPBAR1 activation mechanism

**Fig. 3:**
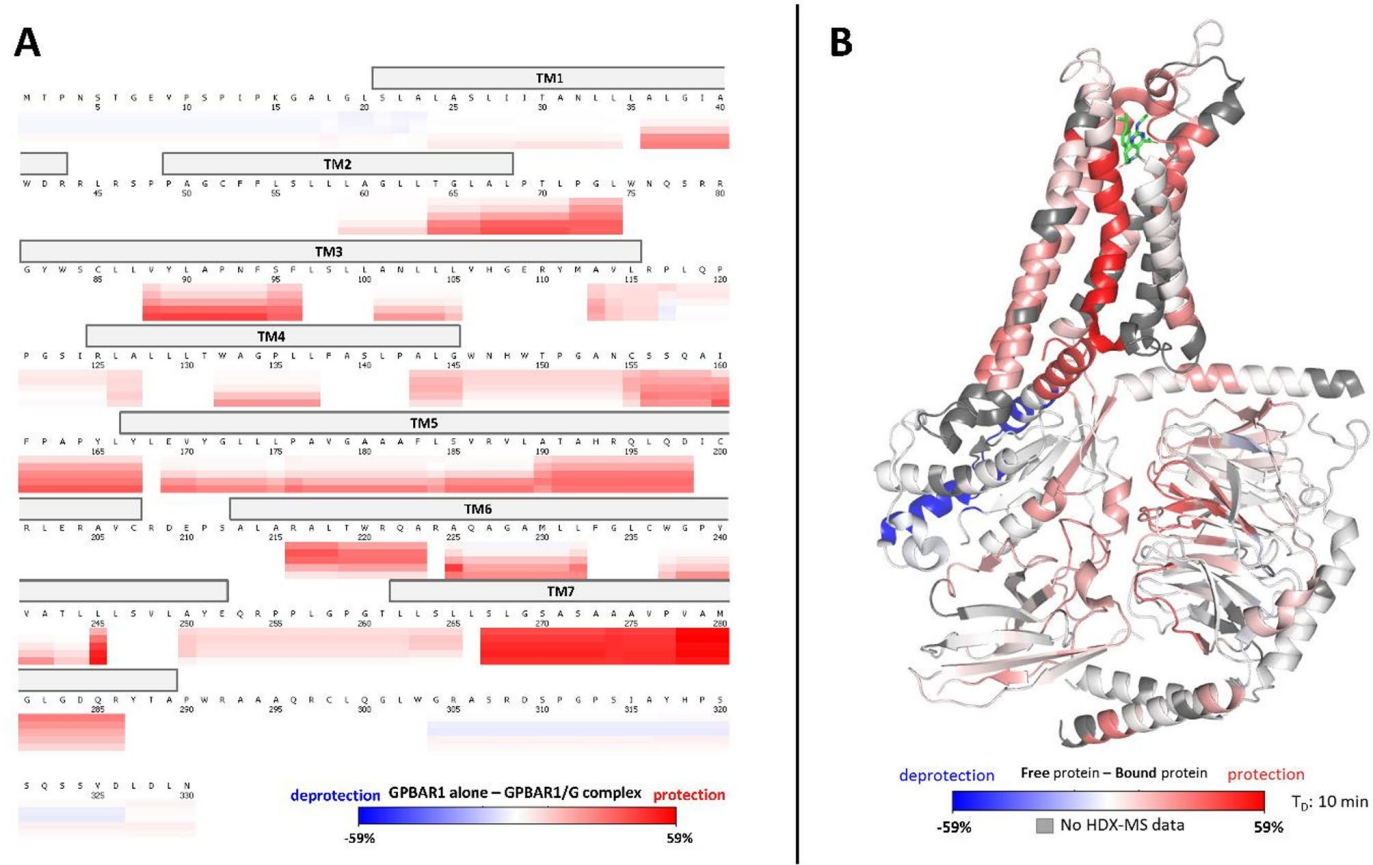
Differential HDX-MS (ΔHDX) of apo GPBAR1 and GPBAR1/Gs complex. (A) Heat map representing the differences in relative D uptake between apo-GPBAR1 alone and complexed GPBAR1/Gs on the amino acid sequence at different deuteration times (5 s, 10 s, 1 min, 10 min, 1 h). (B) Mapping of the HDX-MS data on the GPBAR1/Gs complex cryo-EM structure.

The results of a ΔHDX experiment involving apo-GPBAR1 to holo-GPBAR1/Gs complex were plotted on the 2.5-Å cryo-EM structure (Fig. 3). Almost all regions were observed (including TM2-3 and TM5-7) along the sequence of the GPBAR1 receptor protected from deuteration upon binding to G-protein (Fig. 3.A and supplementary data, Figure S10).

##### Orthosteric P395 ligand binding site

**Fig. 4:**
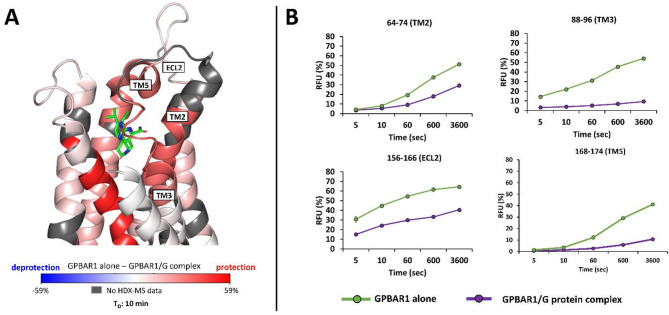
Differential HDX-MS (ΔHDX) of apo GPBAR1 and GPBAR1/Gs complex near the ligand-binding site. (A) Visualization of D incorporation differences (deuteration time: 10 min) on the GPBAR1 structure. Regions are colored according to the given scale (-59%; 59%); those for which no information is available are colored in gray. (B) Relative incorporation in D for 4 peptides from GPBAR1 ligand-binding site obtained in HDX-MS.

Focusing on the known P395 ligand binding region on GPBAR1 (Fig. 4), ΔHDX revealed deuteration in the orthosteric binding pocket of the receptor, reflecting the presence of P395 in the GPBAR1/Gs complex while absent from apo GPBAR1. Several peptides spanning the extracellular part of GPBAR1, the extracellular top of TM5, along with TM2 and TM3 (Fig. 4.B) were significantly protected upon deuteration. Due to their inherent flexibility, the intracellular loops (ICL1-3) are challenging to assess using HDX-MS. Only peptides from ICL2 could be reliably monitored, with peptide 112–125 exhibiting significant protection from D uptake upon GPBAR1/Gs complex formation (Supplementary data, Figure S10). The TM5 helix is highly dynamic (peptides 155–166, 156–166, 156–167, 159–167, 168–173, 168–174), reaching a D incorporation difference of almost 30% after one hour of labeling for peptide 168–174. The TM2 and TM3 helices also exhibit a very high level of protection during complex formation, with some peptides showing 30 to 45% H/D exchange for the longest labeling points (peptides 64–74 or 88–96, for example). These results are in agreement with agonist ligand activation already reported for other class A GPCRs^23,38^. Altogether, the HDX-MS data are consistent with the cryo-EM structure, as all GPBAR1 residues involved in the direct interaction with the P395 ligand in the cryo-EM structure of GPBAR1/G are part of the protected peptides identified by HDX-MS. Inside the orthosteric pocket, a long-stranded hydrophobic strip from TM2 and TM3 holds the tetrahydropyrido[4,3-d] pyrimidine moiety, whereas another hydrophobic patch from TM5 accommodates the 4-isopropylphenyl part of P395 ligand (Fig. 4.A).

Of note, several peptides in the apo GPBAR1 sample exhibit characteristic bimodal isotopic patterns in the region of TM2 (e.g., residues 59–68, 63–71, 64–71), TM3 (e.g., residues 88–96, 89–96, 100–104) and TM6 (e.g., residues 224–232, 225–231, 225–232) (Supplementary data, Figure S11), suggesting that these regions may undergo slow exchange between folded and unfolded conformations. Reports of bimodal distributions in GPCRs remain relatively scarce. EX1 kinetics behaviors have been observed at the C-terminus of the TM6/ECL3 region of the β_2_-adrenergic receptor^39^ and in several regions of the glucagon receptor including the extracellular domain, TM1, TM2 and TM6^40^. Whether our similar observations in GPBAR1 indicate a potential functional link to G protein-induced changes in conformational flexibility or arise from instabilities of GPCR secondary structure during experiments, cannot be definitively ruled out^41^ and might be an interesting field to explore.

##### Almost all transmembrane domains of GPBAR1 are protected from deuteration

**Fig. 5:**
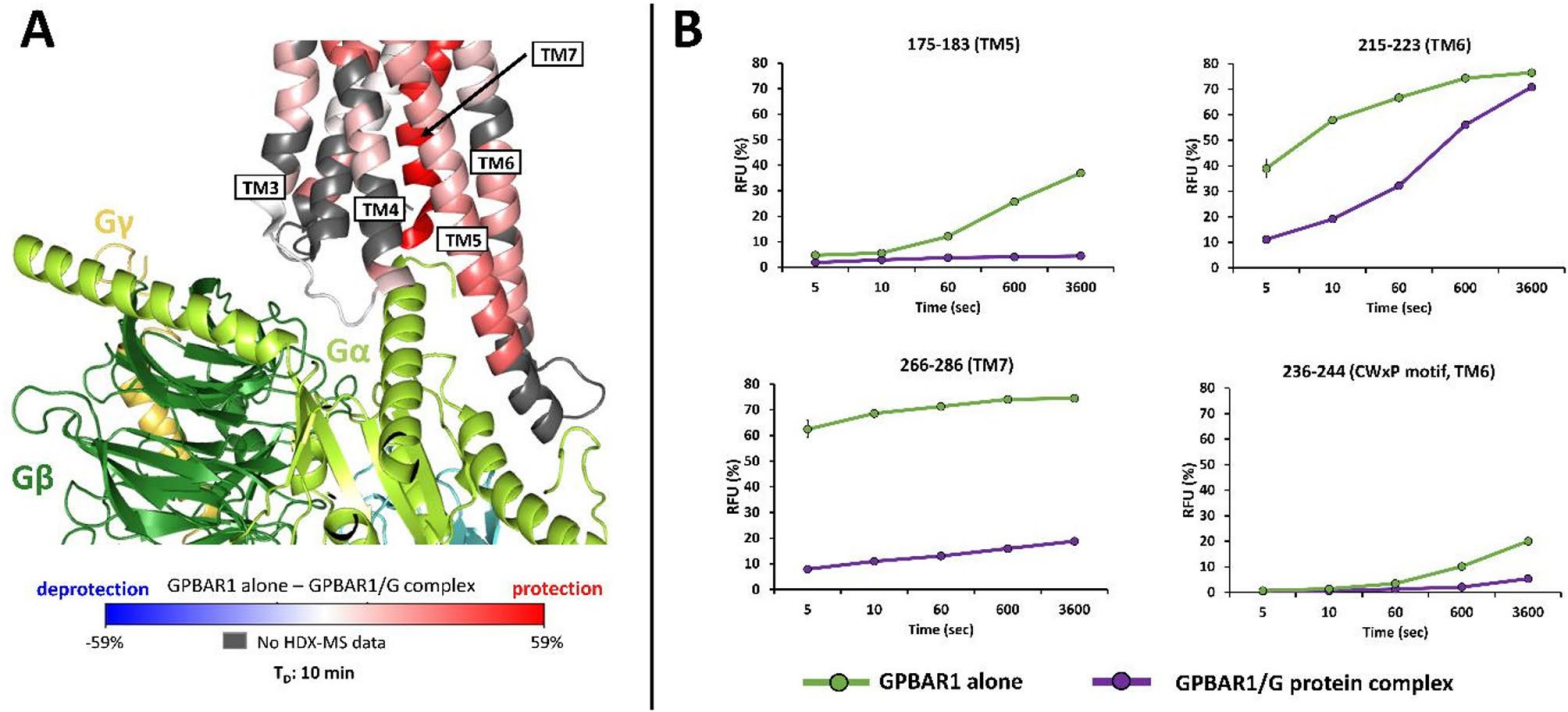
Differential HDX-MS (ΔHDX) of apo GPBAR1 and GPBAR1/Gs complex along the transmembrane domain. (A) Visualization of D incorporation differences (deuteration time: 10 min) on the GPBAR1 structure. Regions are colored according to the given scale (-59%; 59%); those for which no information is available are colored in gray. (B) Relative incorporation in D for 4 peptides from GPBAR1 transmembrane domain obtained in HDX-MS.

Focusing on the TM domains, D incorporation is reduced for TM2, TM3, TM5, TM6, and TM7 in the presence of Gs-trimer, in agreement with the cryo-EM structure (Fig. 5.A). Surprisingly, the largest decrease in D uptake is observed for the TM7 helix, with incorporation differences between 40% and 60% between the two states (Fig. 5.B). We thus conducted a maximally deuterated control (maxD) experiment (Supplementary data, Figure S12) that revealed that helix 7 behaves differently from the other transmembrane helices. Specifically, for TM7, D uptake profiles are very similar to each other, regardless of incubation times, and are especially close to the maxD values^42^ obtained when the protein is denatured. After correction of the back-exchange and a five-second labeling, D uptake already reaches 80% for TM7, compared with an average of 20% for the other transmembrane helices (Supplementary data, Figure S12). It is, therefore, highly likely that the TM7 region is unstructured in the apo form. TM7 helix in GPBAR1 becomes less flexible and forms more contacts with other TM helices upon binding of P395 ligand and Gs proteins (Fig. 4.A).

As a unique characteristic of the activated GPBAR1 is located in the TM5–ICL3–TM6 regions^38^, these were investigated next. In the ΔHDX-MS data set, the TM5 helix is more protected, reaching a D incorporation difference of almost 35% after one hour of labeling (Fig. 5B). These local rearrangements are also accompanied by movements of the intracellular ends of TM5 (peptides 188–198 and 190–198) and TM6 helices. For example, peptide 215–223 from TM6 shows protection against H/D exchange at short time points (5 s to 1 min), whereas no difference is observed at longer time points, suggesting greater exposure of this region to the solvent (Fig. 5B).

Another important region for GPCR activation is the CWxP motif of the ligand-binding pocket found in the TM6 (amino acids at positions 236, 237, and 239 in this case). However, the CWxP motif (peptide 236–244) has very low solvent accessibility (20% incorporation in D after 1 h of labeling), in agreement with the fact that the ligand binding pocket is buried in GPBAR1 (Fig. 5.B).

HDX-MS observations agree with the cryo-EM structure and confirm the general mechanism of GPCR/G interaction for GPBAR1, where Cys236 contributes to the reorganization of TM6-TM7 interactions during receptor activation, while Trp237 movement enables the intracellular end of TM6 to move^43^.

#### Differential HDX-MS (ΔHDX) of G_s_ and GPBAR1/Gs complex support G_s_ conformational remodeling upon complex formation

Because G-trimer alone could not be stabilized and purified without nucleotides while in contrary GPBAR1/Gs could only be obtained in a nucleotide-free environment (and in presence of Nb35 nanobody), we focused this paragraph on different structural regions of Gs and first checked on our HDX-MS dataset for regions expected to be impacted (e.g. GDP binding region, Nb35 epitope, etc.). Of note, in our experimental design we cannot differentiate the conformational change induced upon GDP, Nb35 or GPBAR1 binding. However, we can rely on high resolution crystallographic or cryo-EM structures.

##### Overall conformational changes on the different Gs subunits

The dynamics of the three Gαs, Gβ, Gγ subunits of the G_s_ protein receptor was investigated by ΔHDX comparing GDP-bound-G trimer and GDP-free-GPBAR1/Gs complex (Supplementary data, Figure S13). Significantly different D uptake profiles were observed for the three subunits, with Gαs being the most flexible, Gβ being less affected, and finally Gγ being almost unaffected by GPBAR1 binding (or nucleotide removal).

**Fig. 6:**
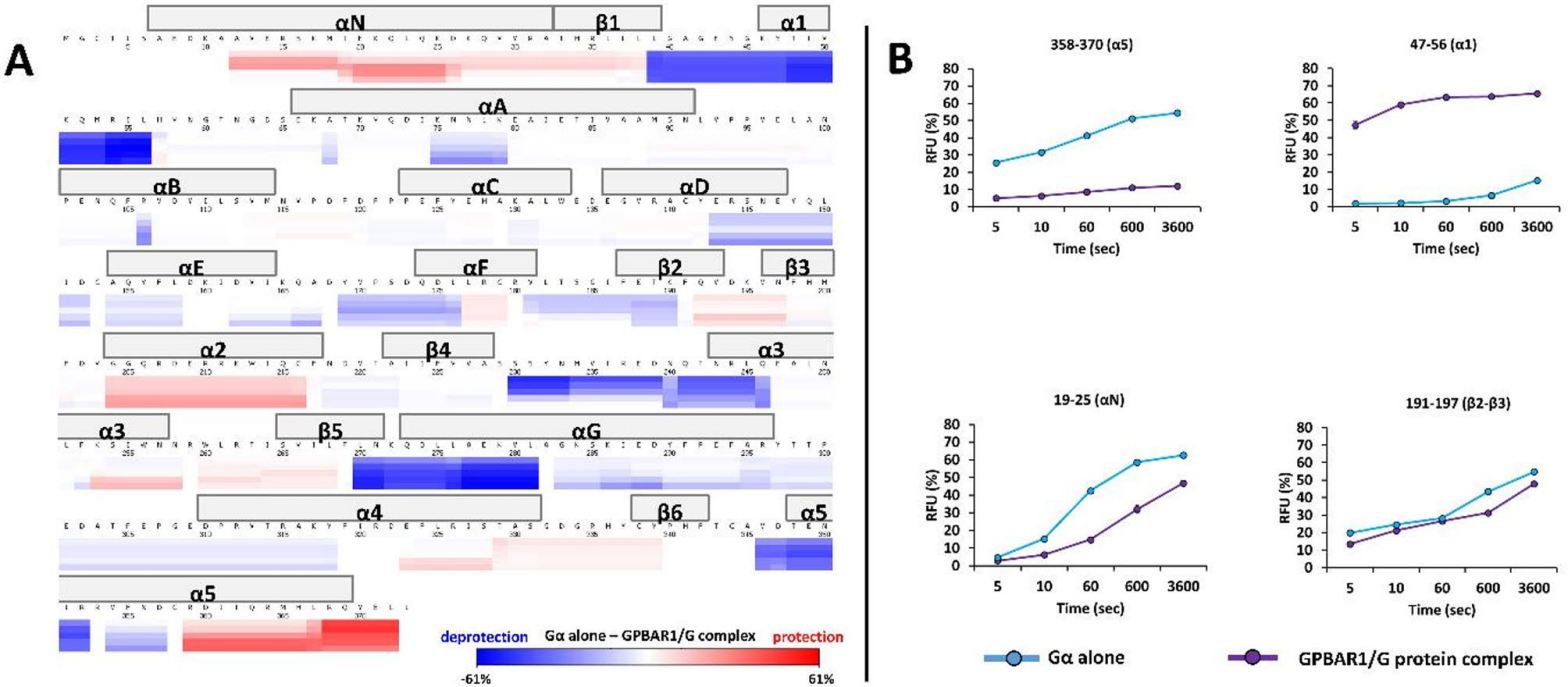
Differential HDX-MS (ΔHDX) of apo GDP-bound Gα and GPBAR1/GDP-free-Gs complex. (A) Heat map representing the differences in relative D uptake between Gα alone and complexed on the amino acid sequence at different deuteration times (5 s, 10 s, 1 min, 10 min, 1 h). (B) Relative incorporation in D for 4 peptides from Gα digestion obtained in HDX-MS.

∆HDX revealed that Gαs is the most impacted, with many regions distributed all over the sequence showing either protection (red) or deprotection (blue) from deuteration (Fig. 6).

In the GPBAR/G_s_ complex, regions of the Gαs GTPase domain became more exposed to the solvent. Helix α1 (e.g. peptide 47–56, Fig. 6.B), as well as regions of α3, αG, and β5, exhibited significantly increased D incorporation after complex formation (50–60% incorporation difference for peptide 47–56). Interestingly, deprotection of peptides 37–54/38–54 from the P-loop and peptide 183–190 from the switch I (see supplementary data, Figure S13) constitutive of the GDP binding site (delimited by three loops: β1-α1 (P-loop), αF-β2 (switch I) and β3- α2 loops^44,45^ were observed. These observations might corroborate the absence of nucleotides in the GPBAR1/Gs complex, triggered by the use of apyrase to favor the complex formation, even if it cannot be definitively ruled out that they might also result, at least partially, from conformational changes induced by the GPBAR1 and/or Nb35 binding to Gα. Other regions of the GTPase domain^38^ involved in GPBAR1 interaction as depicted in the cryo-EM structure, such as αN (peptides 11–18 and 19–25), α2, and α5 helices, regions of α3, α4, β2-β3 (peptide 191–197), and β6 strands (peptide 327–339) were also protected in the HDX-MS dataset.

Insertion of helix α5 is a highly conserved mechanism that is characteristic of the interaction of GPCRs with G proteins^31,32^. In the case of GPBAR receptors, helix α5 insertion is not as deep as that of other class A GPCRs, due to the extension of the TM5 and TM6 helices^38,46^. In our experimental HDX-MS dataset, the C-terminal end of the α5 helix is strongly protected in the GPBAR1/Gs complex, as depicted by the D incorporation profile of peptide 358–370 (end of α5 helix, Fig. 6.B). After one hour of labeling, this peptide reaches a D incorporation difference of nearly 40%. The HDX-MS data also show that the N-terminal part of α5 is more exposed to the solvent and so is not localized inside the receptor, as illustrated by peptide 347–352 that exhibits a D incorporation difference of up to 40%. Altogether HDX-MS observations agree with the cryo-EM structure, revealing that insertion of the α5 helix compacts the structure of GPBAR1 (interaction of residues Gln363, His366, Tyr370 and Leu372 with GPBAR1) and further increases conformational constraints in the extracellular domain where the P395 ligand is located.

Previous studies have shown that the junction between the Gαs GTPase and the helical domain rotate by 127° to allow interaction between the receptor and the rest of the G protein^12^. Overall, the HDX-MS results were consistent with this movement, as peptides peptides 68–78 and 142–150 exhibited increased solvent exposure in GPBAR1/Gs complex (Supplementary data, Figure S13).

##### Conformational changes of the Gβ and Gγ subunits

**Fig. 7:**
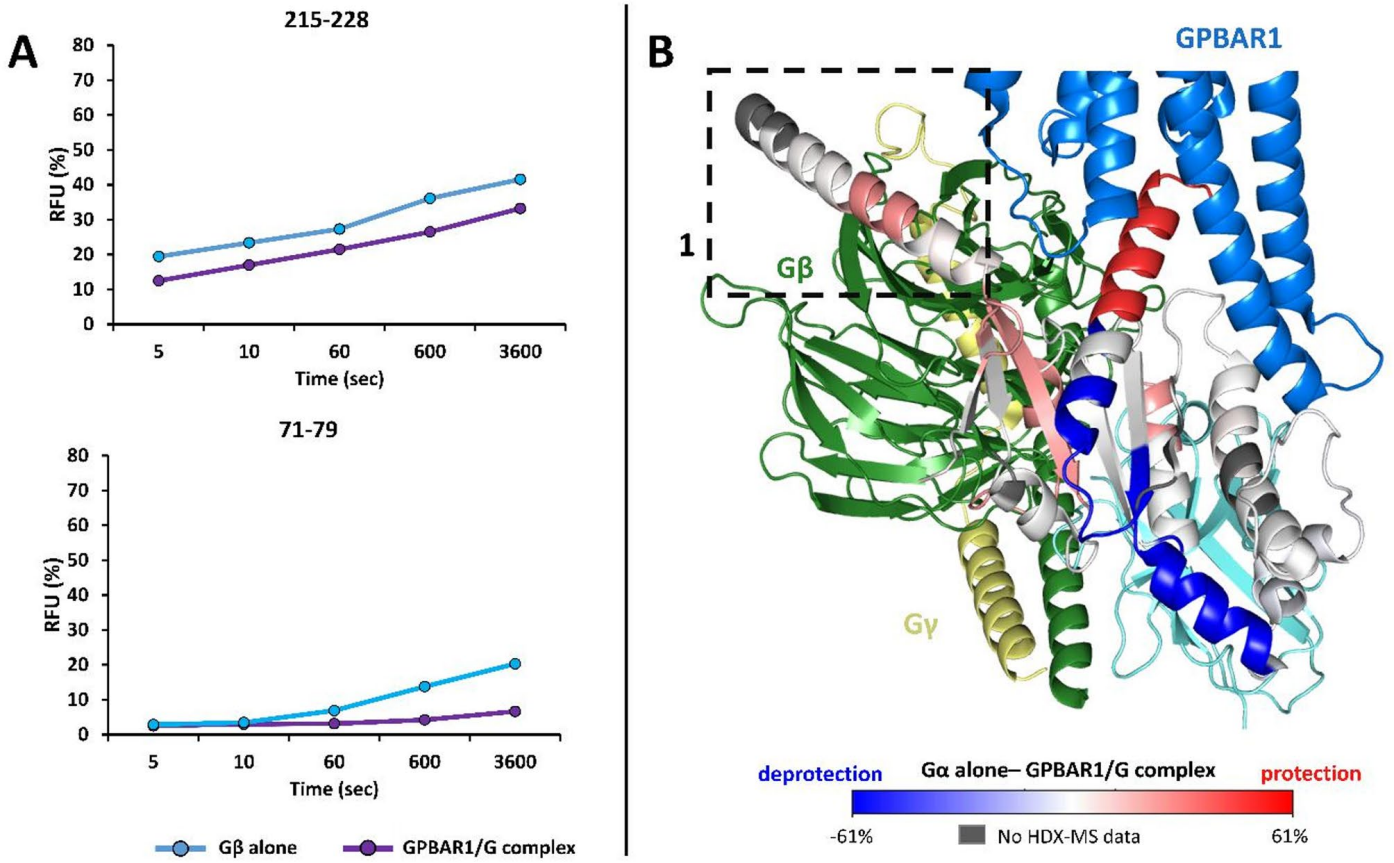
Differential HDX-MS (ΔHDX) of the G_s_ protein and GPBAR1/Gs complex. (A) Relative incorporation in D for 2 peptides from Gβ digestion obtained in HDX-MS. (B) Visualization of incorporation differences on Gαs only. Box 1 shows the interaction between one of the Gαs helices and the Gβ subunit. Visualization of D incorporation differences (deuteration time: 10 min) on the G protein structure. Regions are colored according to the given scale (-61%; 61%); those for which no information is available are colored gray.

Conversely to Gα, fewer regions of the Gβ subunit exhibit protection to deuteration. Lower D incorporation was observed for peptides 71–79, 114–121, and 215–228 in the GPBAR1/Gs complex (Fig. 7). For peptide 215–228 (Fig. 7.A), a significant difference in incorporation and dynamics was observed between the two states, whereas peptide 71–79 (Fig. 7.B and supplementary data, Figure S14) mainly showed only a significant difference in dynamics (Supplementary data, Figure S14). The interaction between Gβ region and the αN helix of the Gαs subunit is depicted in Fig. 7.B. The dynamic effect observed above stems from the fact that the αN helix of Gαs progressively “pushes” part of the Gβ subunit. The cryo-EM structure revealed another interaction interface between Gαs and Gβ subunits, thus explaining the protective effects observed on the α2 helix of Gαs and Gβ. Finally, as expected, no Gγ peptide showed a statistically significant difference in D incorporation (Supplementary data, Figure S13). This result is not surprising, as this small subunit (∼8 kDa) is located at the periphery of the complex and the conformational changes linked to the interaction between GPBAR1 and Gαs are too distant to be visible on Gγ.

## Discussion

A comprehensive description of the molecular determinants and the conformational dynamics of GPBAR1 upon heterotrimeric G_s_ binding was obtained by a combination of mass photometry, HDX-MS, and cryo-EM. Optimization was essential to achieve a high-quality dataset.

This HDX-MS study is one of the few performed on a fully active full-size GPCR (composed of full-length GPBAR1 bound to its agonist P395 ligand) bound to heterotrimeric Gs (comprising Gαs, Gβ, and Gγ subunits, stabilized by Nb35 nanobody and nucleotide-free). Leveraging the complexity of the HDX-MS experiment on a 5-subunit multiprotein membrane protein complex is cutting edge. In addition, achieving sequence coverage > 60% is challenging for membrane proteins, especially for GPCRs. Through an extended optimization of the upstream sample preparation, sequence coverages were > 99% for GPBAR1, resulting in sequence coverages > 75% along with a redundancy > 3.8 in real ΔHDX-MS conditions, allowing a thorough view of the conformational dynamics throughout the GPCR sequence. Cryo-EM generated high-resolution (2.5-Å) structures of the GPBAR1/Gs complex, revealing an additional but previously undescribed highly flexible AH region for GPBAR1.

Since these experiments offer a unique opportunity to compare the dynamic insights provided by HDX-MS with the more static view of cryo-EM, the GPBAR1/Gs study serves as a valuable case study and could be further extended to investigate GPCR/G_s_ protein systems more broadly. Overall, cryo-EM and HDX-MS are highly consistent and corroborate each other, highlighting increased local stability of many intracellular and extracellular regions in GPBAR1 when bound to the G_s_ heterotrimer. This increased stability suggests that the binding of G proteins (stabilized by a nanobody) to the agonist GPBAR1 may reduce the flexibility of specific regions, which could be crucial for receptor activation and signal transduction.

Furthermore, HDX-MS not only reveal conformational rearrangements on the GPBAR1 side but also confirm known hallmarks of conformational changes occurring on the trimeric G_s_. Indeed, HDX-MS confirms that the α5 helix of the Gαs subunit undergoes a significant decrease in H/D exchange upon complex formation. This finding is in good agreement with cryo-EM data, which reveals a direct interaction of the α5 helix with GPBAR1, suggesting that the conformational changes in GPBAR1 upon activation are transmitted through this interaction to the G protein, facilitating signal transduction.

Finally, the complementarity and synergy of atomic resolution by cryo-EM and lower- resolution by HDX-MS and mass photometry using a model GPCR/Gs complex was demonstrated. MPhoto provides a rapid way to monitor the formation of GPCR/Gs complexes in a membrane mimic environment. Despite its lower mass accuracy compared to native MS, MPhoto does not require any prior buffer exchange, limiting sample preparation artifacts, especially with membrane proteins. MPhoto affords assessment of membrane protein homogeneity, oligomeric state, or complex formation within minutes, offering real-time insights into the assembly process and ensuring the quality and integrity of the sample before more complex and time-consuming experiments like HDX-MS and cryo-EM. The systematic combination of cryo-EM and HDX-MS provides valuable information for the analysis of large multiprotein complexes such as GPCR/G protein complexes. While cryo-EM provides a high-resolution, almost static picture of the complex, HDX-MS reveals the dynamic conformational changes that occur during complex assembly and activation. The systematic combination of complementary biophysical techniques provides a comprehensive understanding of the molecular mechanisms underlying GPCR signaling, which could accelerate the development of specific and effective therapeutics.

## Materials and methods

### Mass photometry (MPhoto)

MP measurements were performed on GPBAR receptor and the complex, purified in 20 mM HEPES, 150 mM NaCl, 0.02%/0.002% w/v LMNG/CHS. Analysis were carried out using a TWO^MP^ (Refeyn Ltd, Oxford, UK) at room temperature (18 °C). Samples were first diluted with their native buffer to 100 and 300 nM for apo-GPBAR1 and the GPBAR1/Gs complex, respectively. Finally, 2 µL of the stock solution were drop-diluted and carefully mixed to 10 and 30 nM in a 18 µL Phosphate-buffered saline (PBS) droplet. Three movies of 3000 frames were recorded (60 s) for each sample using the Acquire^MP^ software v2.5.1 (Refeyn Ltd, Oxford, UK). Raw data were processed using the Discover^MP^ software v2.5.0 (Refeyn Ltd, Oxford, UK). Distribution histograms represent the number of counts per contrast value. Contrast values were converted into masses using a contrast-to-mass calibration up to 330 kDa. The distributions of scattering events (given as contrast) were Gaussian-fitted using Discover^MP^.

### Hydrogen deuterium exchange-mass spectrometry (HDX-MS)

#### Peptide identification

Before performing HDX-MS experiments, peptides were identified by digesting undeuterated samples using the same protocol and identical liquid chromatographic (LC) gradient as detailed below and performing mass spectrometry experiments in MS^E^ data acquisition mode with a Xevo G2 XS-QTOF mass spectrometer (Waters, Manchester, UK) over a range of m/z 50–2000. A 0.5 mM sodium formate solution (90/10, 2-propanol/water, v/v) was used for calibration, and a 200 pg/µL leucine enkephalin solution (50/50/0.1, water/ acetonitrile/formic acid, v/v/v) was applied for mass accuracy correction in positive electrospray ionization mode (ESI+) and resolution mode. MS^E^ runs were analyzed using ProteinLynx Global Server (PLGS) 3.0.3 (Waters, Manchester, UK), and peptides identified in each run, with a minimum intensity of 3000, with at least 0.3 fragments per amino acid, 2 consecutive minimum products, and a mass error below 10 ppm were selected in DynamX 3.0 to generate peptides lists (Waters, Manchester, UK).

#### Optimization of the HDX conditions

Prior to labeling experiments, an extensive optimization of GPBAR1 digestion conditions was carried out. All the tested conditions were performed on GPBAR1 samples in duplicate to evaluate the most efficient protocols and optimal parameters to maximize both sequence coverage and redundancy. The injected quantity of GPBAR1 (7, 13, and 26 pmol), the enzyme used for the online digestion (pepsin and nepenthesin II), the nature of the chaotropic/denaturing agent in the quenching buffer, and carryover were successively evaluated. Digestions were performed online on a pepsin-immobilized cartridge (Enzymate pepsin column (300 Å, 5 μm, 2.1 mm × 30 mm, Waters, Manchester, UK) or a nepenthesin II protease column (2.1 mm × 20 mm, Affipro, Czech Republic). The best experimental conditions were employed on the other apo biological partners (G-protein, nanobody, and complex) to ensure the quality of digestion under the same conditions. Subsequently, optimal conditions were used for the total complex. A detailed description of the different buffer compositions tested during the optimization phase is provided in supplementary data (Figures S5 and S7).

####  Labelling experiments

Biological partners – GPBAR1 (26 pmol), trimeric G protein (39 pmol), and nanobody Nb35 (79 pmol) – were deuterated at room temperature alone or in the complex. All analyses were performed using 2% DMSO. Deuteration was carried out by incubating 2 µL of the stock sample solutions into 36 µL of D_2_O buffer (20 mM HEPES pD 7.5, 150 mM NaCl, 0.02% LMNG w/v) for different labeling time points (10 s on ice / 10, 60, 600, and 3600 s at RT), corresponding to a 95% deuterated final solution. 38 µL of each deuterated preparation were quenched with 38 µL of quench buffer (4 M urea pH 2.3, 500 mM TCEP) kept on ice with a final pH of 2.4. Deuteration, quenching, and injection of the samples were manually performed in triplicate.

#### Protein digestion and LC-MS conditions for labeling experiments

Digestion and chromatography were conducted on an Acquity UPLC M-Class System with HDX Technology (Waters, Manchester, UK). Digestion was performed online using a nepenthesin II protease column (2.1 × 20 mm, Affipro, Czech Republic) at a 100 µL/min flow rate of 0.1% formic acid solution at 20 °C for 3 min. Peptides were then trapped on an Acquity BEH C18 VanGuard pre-column (1.7 μm, 2.1 × 5 mm, Waters, Manchester, UK) and separated on an Acquity UPLC BEH C18 analytical column (1.7 μm, 1.0 × 100 mm, Waters, Manchester, UK) at 0.1 °C. Peptide separation was performed at a flow rate of 40 µL/min, with an elution gradient of solvent A (0.1% formic acid, water) and solvent B (0.1% formic acid, acetonitrile) from 5 to 35% B over 7 min followed by a 1 min ramp to 85% B. All exchange reactions were performed in triplicate. To eliminate peptide carryover, the protease column was washed three times between two runs of deuterated samples using alternatively 1.5 M guanidine-HCl in 500 mM glycine buffer (pH 2.3), and a solution composed of 5% acetonitrile, 5% 2-propanol, and 20% acetic acid.

#### HDX-MS data analysis

After a first round of automated spectral processing using DynamX, isotopic profiles for all identified peptides were manually checked and corrected. A back-exchange assessment and related correction were performed for each protein following the protocol described by Peterle et al.^42^ (Supplementary data, Figure S12). MaxD protocol was performed by concentrating 2 µL of each stock sample solution for 5 min with a SpeedVac. The protein material was then resuspended in an equal volume of 7 M Guanidine HCl, H_2_O. After a brief vortex and spin, the protein material was heated at 90 °C for 5 min and then cooled back to room temperature (20 °C). 36 µL of D_2_O buffer (20 mM HEPES pD 7.5, 150 mM NaCl, 0.02% LMNG w/v) were then added to the denaturated protein. The protein solution was heated at 50 °C for 10 min and then cooled back to 20 °C. Finally, each deuterated protein was cooled to 0 °C (ice bath), quenched with 38 µL of quench buffer (4 M urea pH 2.3, 500 mM TCEP) and injected into the mass spectrometer. Differences in deuterium uptake were statistically validated with a p-value of 0.01 with the MEMHDX software^47^ with statistical significance thresholds (Supplementary data, Figure S14).

### Cryogenic electron microscopy (cryo-EM)

####  Grid preparation and data acquisition

4 µL of sample were applied onto an UltrAuFoil grid (Quantifoil R1.2/1.3, 300 mesh), which was rendered hydrophilic by a 90 s treatment in an ELMO glow discharge system operating at 3 mA and in a partial vacuum of 3.6 × 10^− 1^ mbar. The grid was then blotted for 10 s at a blot force of 15 and flash-frozen in liquid ethane using a Vitrobot Mark IV (Thermo Fisher Scientific) at 6.5 °C and 100% humidity. Images were acquired on a Titan Krios G4 (Thermo Fisher Scientific) operating at 300 kV in nanoprobe mode using SerialEM software version 4.1.0 beta for automated data collection. Movies were recorded on a Falcon 4 direct electron camera after a SelectrisX energy filter using a 10 eV slit width. Images were collected at a magnification of 165,000x (corresponding to a pixel size of 0.731 Å). The defocus range was − 0.8 to -1.8 μm. Each movie was composed of 703 frames with a total dose of 50 e/Å^2^. The frames were regrouped into 39 fractions, and the first fraction was excluded during motion correction.

#### Data processing

All data processing was performed in RELION version 4.0. Figure S3 in the supplementary data provides a detailed overview of the data processing workflow. The alignment of movie frames, dose weighting, and correction of beam-induced specimen motion were done using RELION’s implementation of MotionCorr. Contrast Transfer Function (CTF) estimation was performed using ctffind4. Picking of particles was carried out using the RELION Gaussian blob picker. Initially, particles were extracted and binned four times to speed up the initial data analysis step. Three rounds of 2D classification were performed to remove images containing damaged or aggregated particles and ice contamination, followed by 3D classification with alignment. The 3D class that best represented our complex, showing high-resolution structural features, was chosen for further analysis and particles were re-extracted at their original size to perform refinement. The obtained alignment was used to perform 3D classifications without alignment, one was with no mask and T = 10 to keep the best particles for high-resolution refinement, and the second with a mask and T = 50 to classify the flexible region of G alpha. Selected classes from each classification were refined using RELION auto-refine job. Ctf refinement and particle polishing were performed. EM Reayd was used for map sharpening.

#### Model building

Structure 7CFM was fitted in cryo-EM map and modified using coot version 0.9.8.2. Geometry optimization was performed with servalcat, and final refinement using Refmac servalcat. The map used for refinement was a normal post-process map from RELION.

### Protein production

#### Construction and expression of GPBAR1

The gene for human full-length GPBAR1 (UniProtKB-Q8TDU6) was cloned into pFastBac vector. To facilitate protein expression and subsequent purification, a HA signal peptide followed by a FLAG epitope, a 3 C protease site, and thermostabilized apocytochrome b562RIL (BRIL) were added at the N-terminus of GPBAR1. The construct was expressed in Sf21 insect cells using the Bac-to-Bac baculovirus expression system (Thermo Fisher Scientific). The cells were grown in SFM Sf-900 II medium (Gibco) to a density of 4.5 × 10^6^ cells/mL and infected with the recombinant baculovirus at a multiplicity of infection of 2 at 27 ℃ for 48 h. Cells were then harvested by centrifugation and stored at -80 °C for future use.

#### GPBAR1 purification

The cell pellets were thawed and lysed by osmotic shock in 10 mM Tris-HCl pH 8, 1 mM EDTA buffer containing iodoacetamide (2 mg/mL), and complete Protease Inhibitor Cocktail Tablets (Roche). After centrifugation (15 min at 38,000 g), the membranes were solubilized using a glass dounce tissue grinder in a solubilization buffer containing 20 mM HEPES pH 7.5, 100 mM NaCl, 1% lauryl maltose neopentyl glycol (LMNG; Anatrace), 0.1% cholesteryl hemisuccinate Tris salt (CHS; Anatrace) supplemented with 2 mg/mL iodoacetamide and complete Protease Inhibitor Cocktail Tablets (Roche). The extraction mixture was stirred at 4 °C for 1 h. The cleared supernatant (38,000 g centrifugation) was loaded by gravity flow onto anti-Flag M2 antibody resin (Sigma-Aldrich). The resin was then washed with 20 column volumes of a wash buffer composed of 20 mM HEPES pH 7.5, 150 mM NaCl, 0.06% LMNG, and 0.006% CHS. The bound protein was eluted in the wash buffer supplemented with 0.2 mg/mL Flag peptide (Sigma-Aldrich). The eluted protein was concentrated to 500 µL using a 50 kDa spin filter and further purified by size exclusion chromatography on a Superdex 200 Increase 10/300 column (Cytiva) in a buffer containing 20 mM HEPES pH 7.5; 150 mM NaCl, 0.006% LMNG; 0.0006% CHS. The fractions corresponding to mostly monodisperse protein were collected, concentrated with a 50 kDa spin filter, and subjected to a second SEC on a Superose 6 Increase 10/300 column (Cytiva) with a buffer containing 20 mM HEPES pH 7.5; 150 mM NaCl, 0.006% LMNG; 0.0006% CHS to reach a better separation from oligomeric or aggregated species. The fractions containing monodisperse protein were pooled and concentrated to 3.6 mg/mL for HDX-MS experiments.

#### Construction, expression, and purification of G_s_ heterotrimer

Gs heterotrimer was expressed in Sf21 cells grown in SFM Sf-900 II medium (Gibco). Human Gαs subunit with 1 to 15 original residues swapped with 1 to 18 residues of Gαi (Kawai et al., 2020) was cloned in pFastBac vector, while N-terminal 6×His-tagged human Gβ1, and human Gγ2 were cloned into pFastBac-Dual vector. The baculoviruses were generated and amplified using the Bac-to-Bac baculovirus expression system (Thermo Fisher Scientific). Sf21 cells cultured in SFM Sf-900 II medium (Gibco), at a density of 4 × 10^6^ cells/ml, were coinfected with both viruses at a 1:1 Gαs: Gβ1γ2 ratio for 48 h at 28 °C. Cells were harvested by centrifugation and pellets were stored at − 80 °C. Cells were resuspended in lysis buffer (10 mM Tris pH 7.4, 100 µM MgCl_2_, 5 mM β-mercaptoethanol (β-ME), 10 µM guanosine diphosphate (GDP) and complete Protease Inhibitor Cocktail Tablets (Roche)) until pellets thawed. The lysate was spun for 15 min at 38,000 g, and then pellet was homogenized in solubilization buffer (20 mM HEPES pH 7.5, 100 mM NaCl, 1% sodium cholate, 0.05% LMNG, 5 mM MgCl_2_, 5 mM β-ME, 10 µM GDP, 5 mM imidazole, and complete Protease Inhibitor Cocktail Tablets (Roche)) using a glass tissue grinder. Solubilization further proceeded for 1 h with gentle stirring at 4 °C before insoluble debris was removed by centrifugation at 38,000 g for 30 min. Pre-equilibrated nickel-bound Superflow resin (TaKaRa) was added to solubilized supernatant and the mixture was incubated on rotation device for another 1 h at 4 °C. The Gs-bound resin was collected by centrifugation for 5 min at 4,000 g and transferred to a gravity column. Beads were washed with 10 CV of the wash buffers containing increasing concentrations of LMNG and decreasing concentrations of cholate until a final wash in 50 mM NaCl, 20 mM HEPES pH 7.5, 0.1% LMNG, 1 mM MgCl_2_, 5 mM β-ME, 100 µM GDP and 20 mM imidazole, before being eluted with the same buffer containing 200 mM imidazole. Then 1 µL antarctic phosphatase (NEB) was added and the sample incubated overnight at 4 °C. The next day the eluate was diluted two-fold with 50 mM NaCl, 20 mM HEPES pH 7.5, 0.1% LMNG, 1 mM MgCl_2_, 5 mM β-ME, 100 µM GDP to decrease imidazole concentration, passed through a 0.22-µm filter, and loaded onto a pre-equilibrated Q Sepharose resin. The resin was washed with 10 CV of wash buffer (20 mM HEPES pH 7.5, 100 mM NaCl, 0.02% LMNG, 1 mM MgCl_2_, 100 µM Tris(2-carboxyethyl)phosphine (TCEP), 10 µM GDP), and the bound protein was eluted in the wash buffer containing 250 mM NaCl. The fractions containing Gs heterotrimer were pooled, concentrated to 1–2 mL, and diluted three-fold in 20 mM HEPES (pH 7.5, 0.02% LMNG, 1 mM MgCl_2_, 100 µM TCEP, 10 µM GDP) in a drop-wise manner to set the final concentration of NaCl to 125 mM. Gs heterotrimer concentrated to 11 mg/mL was flash-frozen and stored at -80 °C for HDX-MS experiments.

#### Nb35 expression and purification

Nanobody-35 (Nb35) bearing a C-terminal 6His-tag was expressed in the periplasm of *Escherichia coli* strain BL21 (DE3) and grown in a TB culture medium with 100 µg/mL ampicillin at 37 °C, 200 rpm. When OD600 reached 1, 0.7 mM IPTG was added to induce its expression. Induced cultures were grown overnight at 18 °C. The cells were collected by centrifugation and lysed in 20 mM Tris-HCl pH 7,5; 150 mM NaCl; 20% Glycerol; EDTA-free Protease Inhibitor Cocktail tablets (Roche) by sonication. After lysis, cell fragments were removed by centrifugation. The supernatant was collected and incubated for 2 h with Ni-NTA beads on a rotation device at 4 °C. The beads were collected and washed with a buffer composed of 50 mM Tris pH8, 500 mM NaCl, 20 mM imidazole. The protein was eluted in the wash buffer supplemented with 200 mM imidazole. The eluate was concentrated with 5 kDa spin concentrator and loaded on a Superdex 200 Increase 10/300 column (GE Healthcare) using 20 mM HEPES pH 7.5, and 150 mM NaCl as a buffer. Fractions containing the monodisperse peak of Nb35 were pooled, concentrated with a 5-kDa centrifugal filter concentrator, and flash-frozen in liquid nitrogen for later use.

#### Expression and purification of GPBAR1/Gs complex

Sf21 cells were cultured in SFM Sf-900 II medium (Gibco) at 28 °C to a density of 4.5 × 106 cells/ml and then were co-infected with GPBAR1, Gαs and Gβ1γ2 using Bac-to-Bac baculovirus expression system (Thermo Fisher Scientific) at the ratio of 1:1:1. The cells were collected by centrifugation 48 h post-infection and stored at -80 °C until use.

Cell pellets from 2 L of culture were resuspended in 30 mM Hepes pH 7.5; 100 mM NaCl; 10 mM MgCl_2_, 0.2 mM TCEP, supplemented with complete Protease Inhibitor Cocktail tablets (Roche). GAPBR/Gs/Nb35 complex formation was initiated by the addition of 20 µM P395, Nb35 (10 µg/mL) and apyrase (25 mU/mL, Sigma-Aldrich). The suspension was incubated for 1.5 h at room temperature and the membrane pellets were collected by centrifugation at 38,000 g for 30 min. The membranes were solubilized using a glass dounce tissue grinder in a solubilization buffer containing 20 mM HEPES pH 7.5, 100 mM NaCl, 10 mM P395, 1% LMNG, 0.1% CHS, and complete Protease Inhibitor Cocktail Tablets (Roche). The sample was further incubated for 1 h with gentle stirring at 4 °C. The supernatant was collected by centrifugation at 38,000 g for 30 min and then incubated with 5 mL Talon resin for 1 h at 4 °C. The resin was extensively washed with a wash buffer containing 20 mM HEPES (pH 7.5; 150 mM NaCl, 0.06% LMNG; 0.006% CHS; 0.02% GDN; 2 mM MgCl_2_, 5 µM P395; 100 µM TCEP) and the bound proteins eluted in the same buffer supplemented with 200 mM imidazole. Then the eluate was slowly loaded on anti-Flag M2 antibody affinity resin, washed with a buffer composed of 20 mM HEPES (pH 7.5; 150 mM NaCl, 0.006% LMNG; 0.0006% CHS; 0.02% glyco-diosgenin (GDN; Anatrace); 2 mM MgCl_2_, 5 µM P395; 100 µM TCEP) and eluted in the same buffer supplemented with 0.2 mg/mL Flag peptide. The eluted complex was concentrated and further purified by size-exclusion chromatography using a Superose 6 Increase 10/300 column (GE Healthcare) pre-equilibrated with buffer containing 20 mM HEPES (pH 7.5; 150 mM NaCl, 0.006% LMNG; 0.0006% CHS; 0.02% GDN; 2 mM MgCl_2_, 5 µM P395; 100 µM TCEP). Eluted fractions that consisted of GPBAR1/G_s_/Nb35 complex were pooled and concentrated with a 100-kDa centrifugal filter concentrator to 4 mg/mL. The obtained complex was either directly used to prepare cryo-EM grids or flash-frozen in liquid nitrogen for HDX-MS experiments.

## Supplementary Information

Below is the link to the electronic supplementary material.

Supplementary Material 1

## Data Availability

The PDB model and the cryo-EM map have been deposited in the Protein Data Bank and the Electron Microscopy Data Bank, and are accessible using these identifiers PDB: 9GYO, EMD-51700. The HDX-MS data have been deposited to the ProteomeXchange Consortium via the PRIDE partner repository with the dataset identifiers XD047822, PXD047827 and PXD04783. Research data will be made available upon request to sarah.cianferani@unistra.fr.

## Abbreviations

Cryo-EM: cryogenic electron microscopy
HDX-MS: hydrogen-deuterium exchange mass spectrometry
GPBAR1: G Protein-Coupled Bile Acid Receptor 1
