# Supplementary material for: Deciphering molecular determinants of GPBAR1-Gs protein interactions by HDX-MS and cryo-EM
Source: Sci Rep. 2025 Aug 26;15:31517. doi: 10.1038/s41598-025-16529-w (PMC12381156; doi:10.1038/s41598-025-16529-w)
Supplement: Supplementary file 1 — Supplementary Material 1 [file 41598_2025_16529_MOESM1_ESM.docx]

**SUPPLEMENTARY DATA**

**Deciphering molecular determinants of GPBAR1-Gs protein interactions by HDX-MS and CryoEM**

**Authors:** Jérôme CASTEL^*1,2^, Thomas BOTZANOWSKI^*1^, Ieva BROOKS^3^, Alexandre FRECHARD^3^, Gilbert BEY^3^, Marine SCHROETER^3^, Elise DEL NERO^1^, François DEBAENE^1^, Fabrice CIESIELSKI^3^, Denis ZEYER^1,3^, Sarah CIANFERANI^2#^ and Renaud MORALES^1#.^

**Affiliations:**

1 - NovAliX, Department of Biophysics, 16 Rue d’Ankara, 67000, Strasbourg, France

2 - Laboratoire de Spectrométrie de Masse BioOrganique, IPHC UMR 7178, Université de Strasbourg, CNRS, Strasbourg 67087, France; Infrastructure Nationale de Protéomique ProFI, UAR2048 CNRS CEA, Strasbourg 67087, France

3 - NovAliX, Department of Structural Biology, 16 Rue d’Ankara, 67000, Strasbourg, France

* contributed equally

**# corresponding authors:** Sarah CIANFERANI : Laboratoire de Spectrométrie de Masse BioOrganique, IPHC UMR 7178, Université de Strasbourg, CNRS, Strasbourg 67087, France - Telephone number: +33368852679 - email address: sarah.cianferani@unistra.fr; Renaud Morales: NovAliX, Department of Biophysics, 16 Rue d’Ankara, 67000, Strasbourg, France. Telephone number:+33367341172; email address: rmorales@novalix.com

- **Protein production**


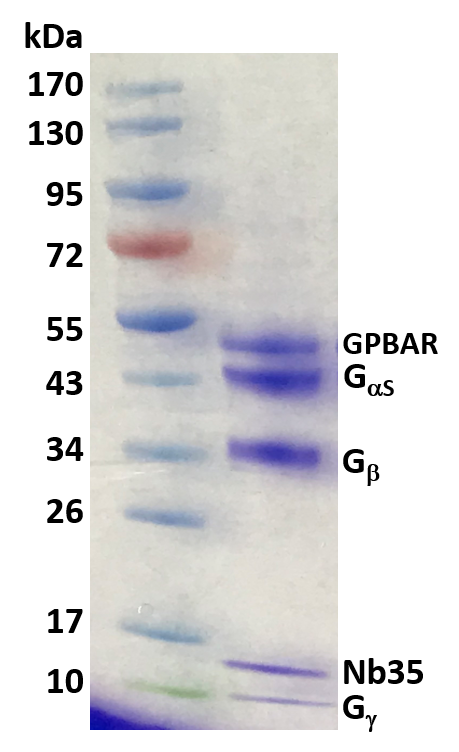
 **
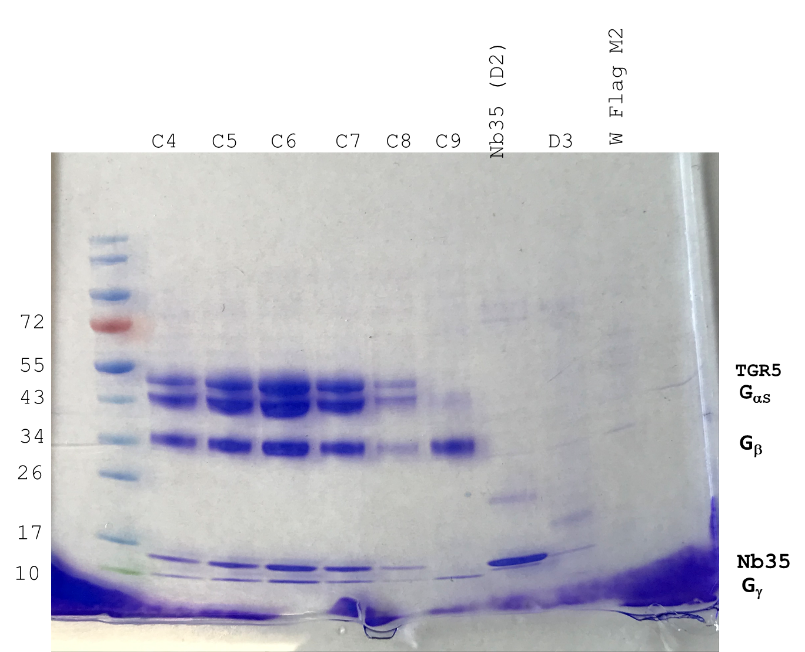
**

Figure S1: 1D-gel SDS-PAGE of the GPBAR1/G protein complex. Left Panel : zoom on Lane C4 ; right Panel : entire annotated gel.

- **Reproducibility of MPhoto experiments**


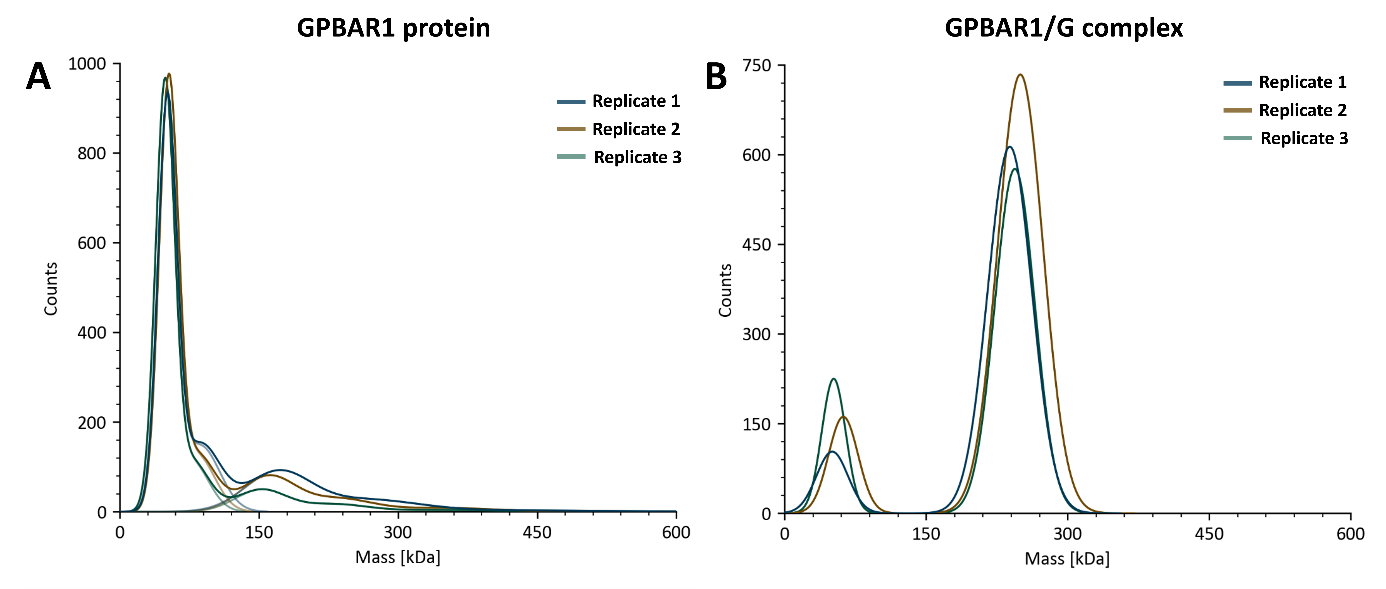


Figure S2: Assessment of MPhoto experiment reproducibility. Superposition of analyses of three GPBAR1 (A) and GPBAR1/G protein complex (B) technical replicates. The continuous curve corresponds to the mass distributions of the majority of species fitted with a Gaussian function.

- **CryoEM data processing method**


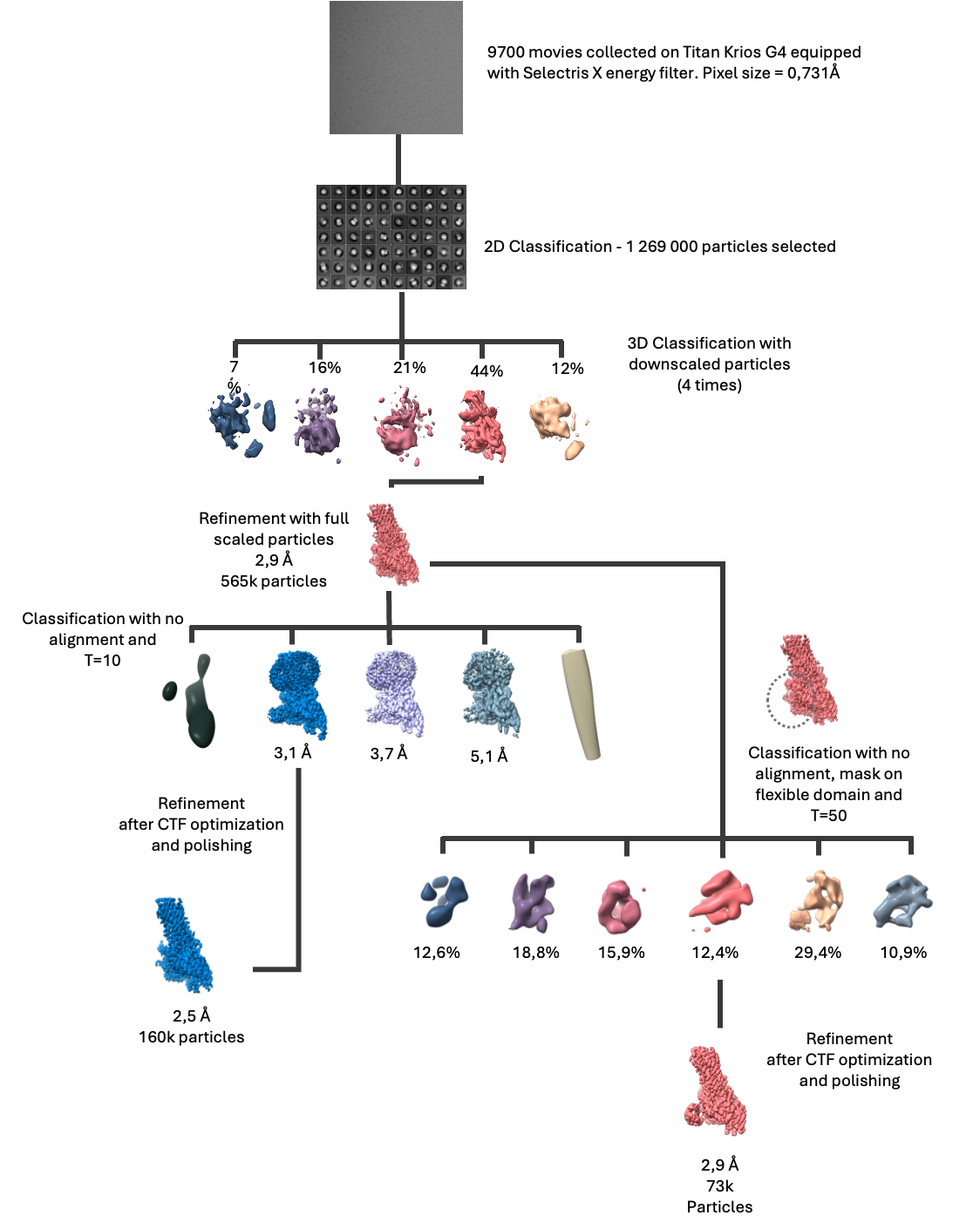


Figure S3: Cryo-EM data processing. Schematic diagram of cryo-EM data analysis, showing how cryo-EM maps were obtained. 9700 micrographs were collected. After initial 3D classification, 1,269,000 were selected and classified in 3D. The resulting particles then underwent further 3D classification to refine two high-resolution cryo-EM maps.


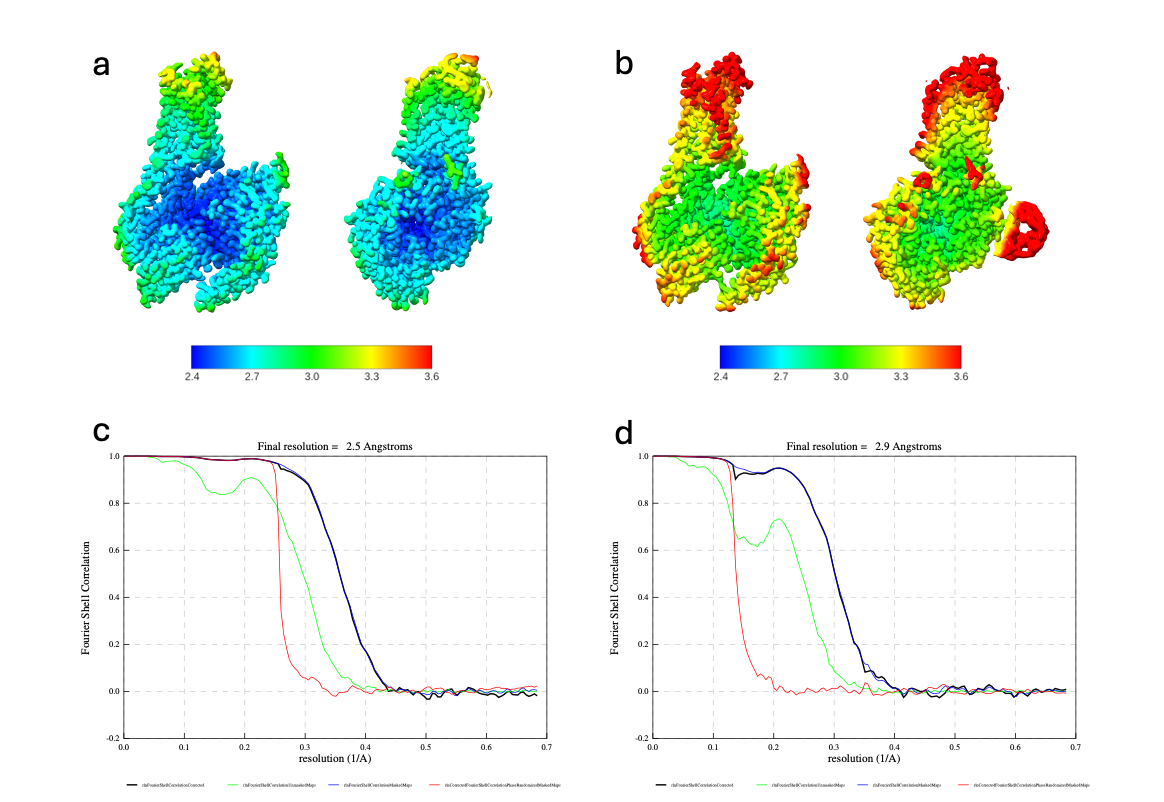


Figure S4: Local and global resolution map estimation. (a,b) Local resolution of the two cryo-EM maps obtained, to compare their resolutions, the same color scale has been used. (c,d) FSC curve of the structures obtained.

- **Optimization of protein digestion**


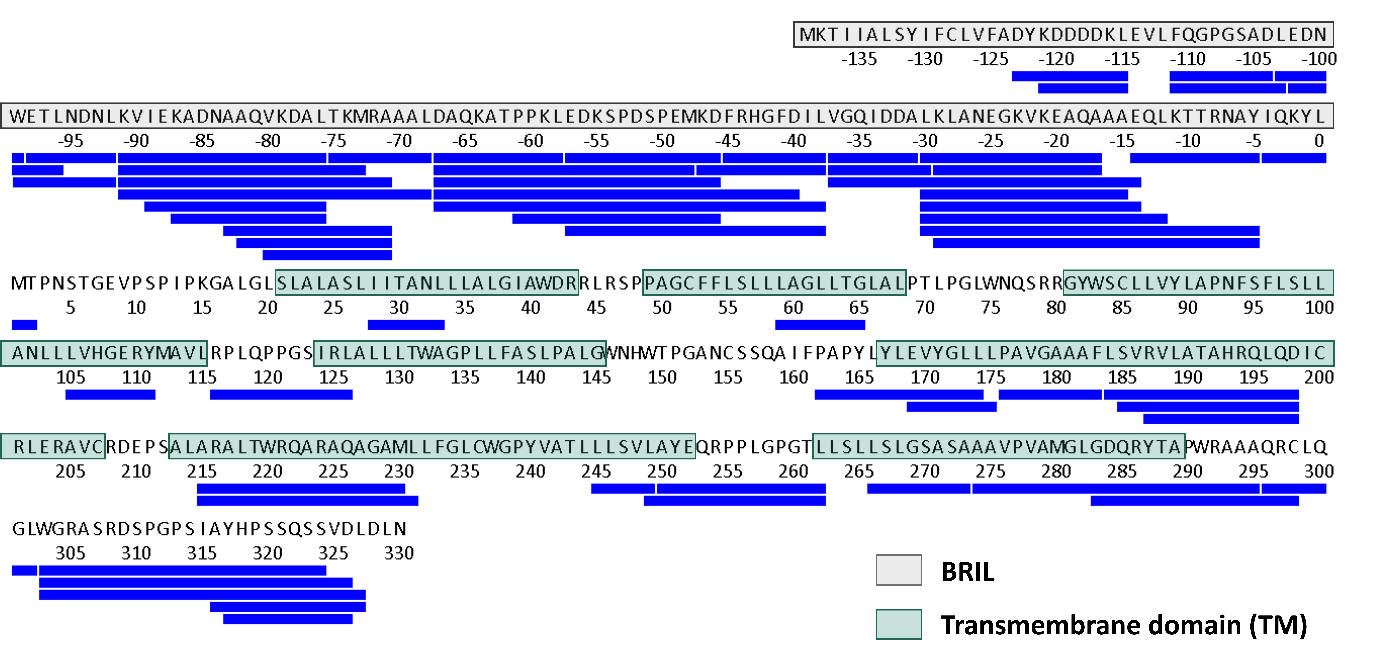


Figure S5: Presentation of peptides (blue) identified after pepsin digestion of the apo GPBAR1 sample before HDX-MS optimizations. 7 pmoles were injected. A Q buffer consisting of 2 M guanidine-HCl was used. Sequence coverage: 61.3%. Number of peptides identified: 64. Redundancy: 3.1.


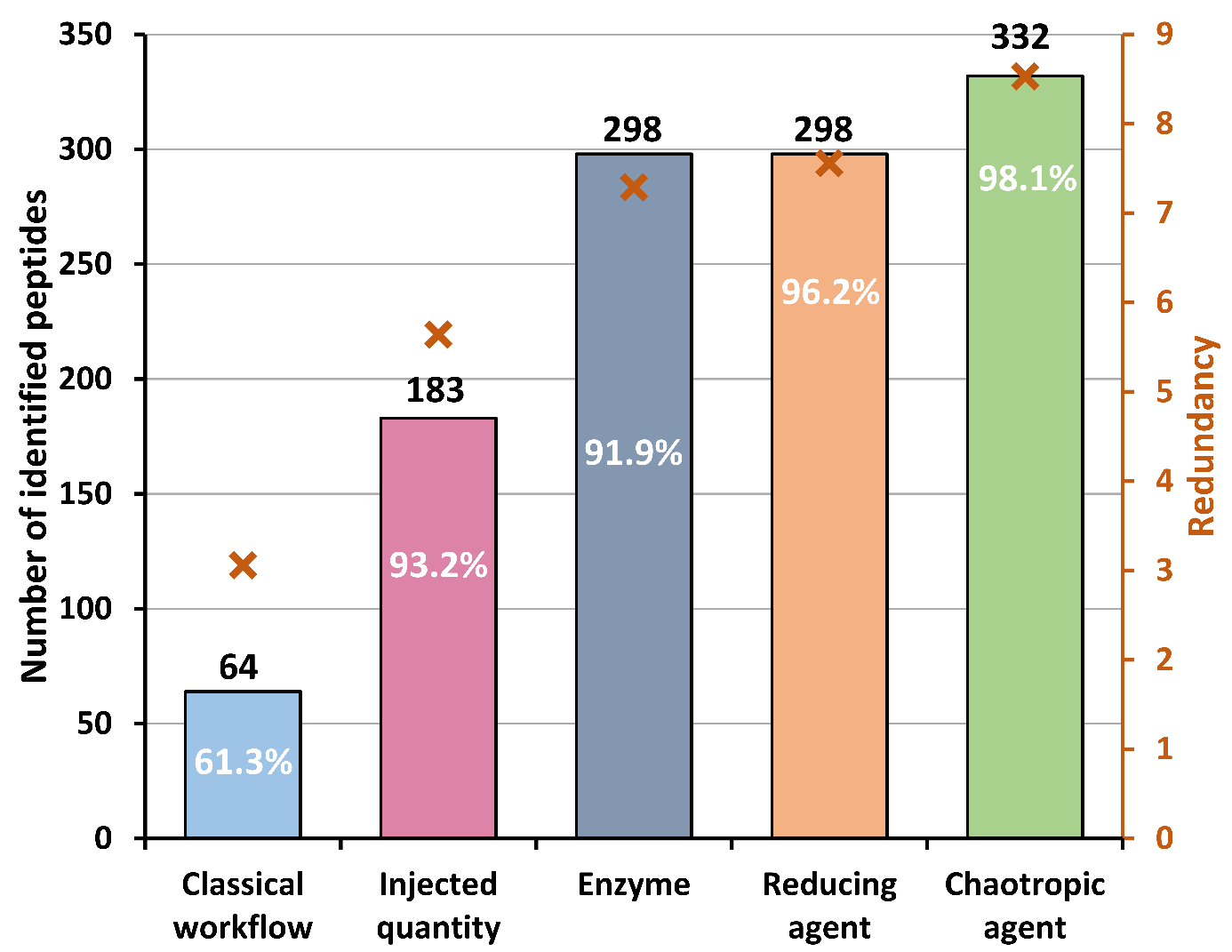


Figure S6: Importance of protein digestion optimization on identified peptides, and the redundancy. Redundancy: Description of how often an amino acid is covered by peptides calculated by DynamX version 3.0 for residues making up the sequence coverage. Number of identified peptides: peptides consisting of between 5 and 30 amino acids. The peptides had a mass error below 10 ppm with at least 0.3 identiﬁed fragments per amino acid in two out of two MS/MS ﬁles. The influence of key experimental parameters is shown on the graph. **Classical workflow**: 7 pmoles of GPBAR1 were injected on a pepsin column, and a quench buffer consisting of Guanidine HCl 2M was used. Sequence coverage: 61.3%. Redundancy: 3.1. **Injected quantity:** 26 pmoles of GPBAR1 were injected on a pepsin column, and a quench buffer consisting of Guanidine HCl 2M was used. Sequence coverage: 93.2%. Redundancy: 5.4. **Enzyme:** 26 pmoles of GPBAR1 were injected on a nepenthesin II column, and a quench buffer consisting of Guanidine HCl 2M was used. Sequence coverage: 91.9%. Redundancy: 7.3. **Reducing agent:** 26 pmoles of GPBAR1 were injected on a nep. II column, and a quench buffer consisting of Guanidine HCl 2M TCEP 500 mM was used. Sequence coverage: 96.2%. Redundancy: 7.6. **Chaotropic agent:** 26 pmoles of GPBAR1 were injected on a nep. II column, and a quench buffer consisting of Urea 4M TCEP 500 mM was used. Sequence coverage: 98.1%. Redundancy: 8.5.

Table S1: Summary of HDX-MS experimental conditions for the study of the GPBAR1/G_S_ complex.

| Injected amount (pmoles) | 26 (GPBAR1) ; 39 (G_s_) ; 79 (Nb35) |
| --- | --- |
| Dilution buffer | 20 mM HEPES pH 7,5 150 mM NaCl 0,02% LMNG m/v |
| Quench buffer | Urée 4 M TCEP 500 mM pH 2,40 |
| Enzyme | Nepenthesine II |


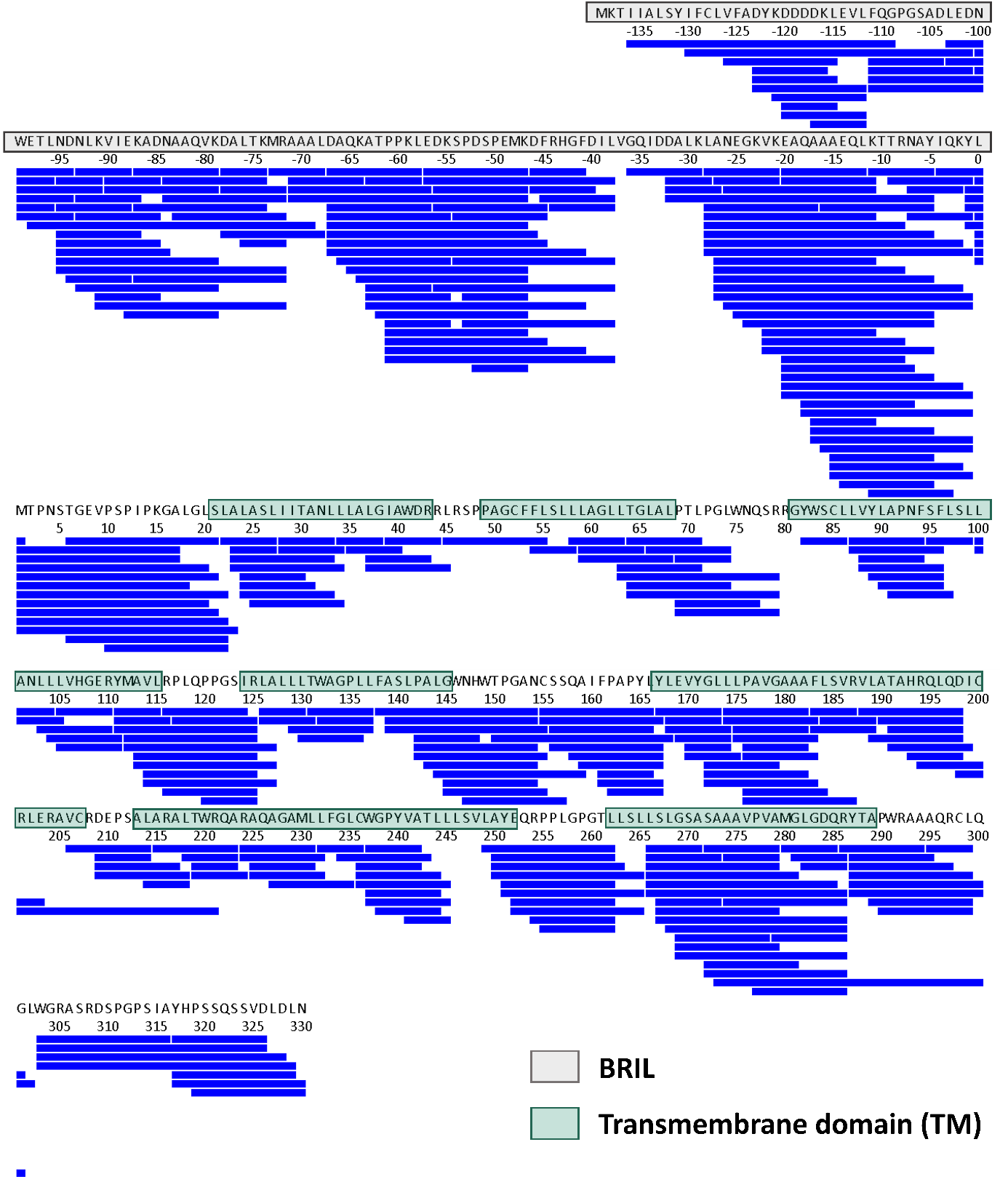


Figure S7: Presentation of peptides (blue) identified after nepenthesin II digestion of the apo GPBAR1 sample. 26 pmoles were injected, and a Q buffer consisting of 4 M urea and 500 mM TCEP was used. Sequence coverage: 98.1%. Number of peptides identified: 332. Redundancy: 8.5.

Table S2: Impact of the manual data curation on the number of valuable GPBAR1 peptides used for data interpretation. Analysis were carried out in triplicates for each protein state (file threshold: 6/6).

| **Manual data curation** | **Before** | **After** |
| --- | --- | --- |
| **Sequence coverage** (%) | 79.4 | 76.4 |
| **Number of peptides** | 160 | 117 |
| **Redundancy** | 5.4 | 3.8 |

- **Nanobody Nb35, as control for our HDX-MS experiments**


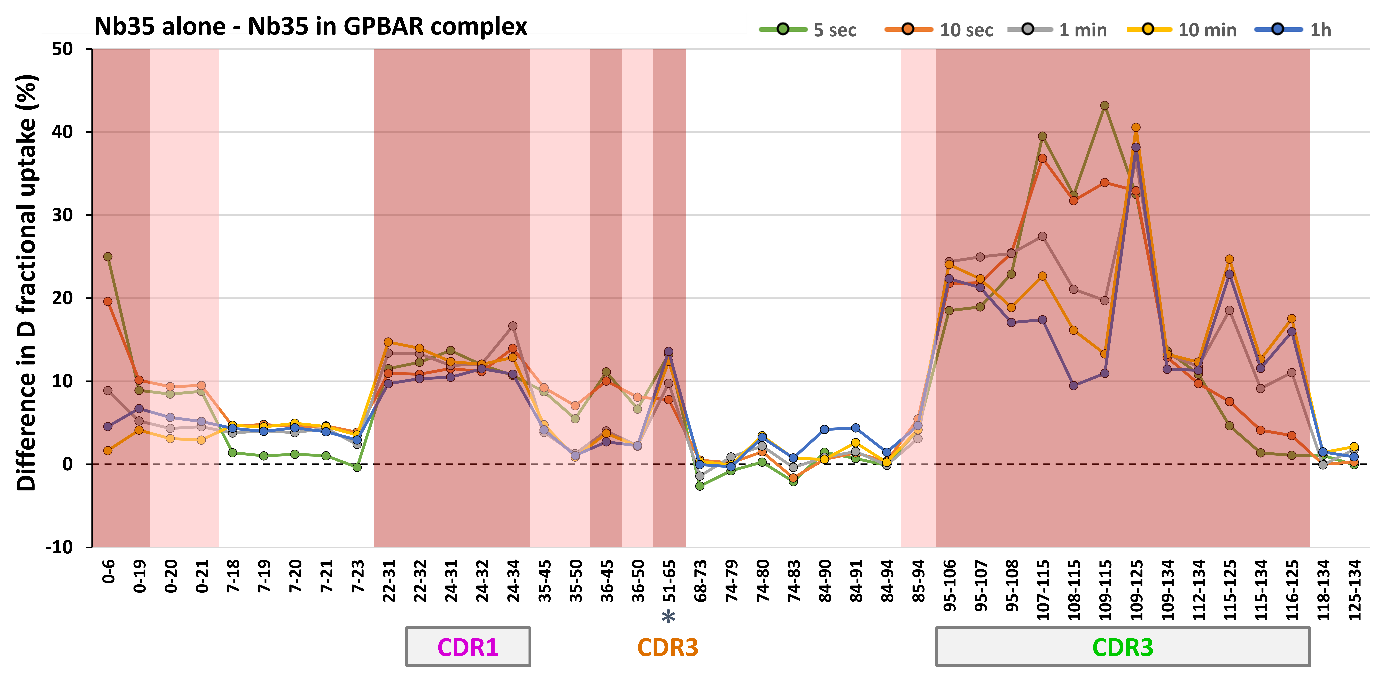


Figure S8: Differences in relative D uptake between Nb35 alone and complexed in GPBAR1/Gs for each peptide identified at different deuteration times. Boxed peptides show statistically significant differences in D incorporation (MEMHDX, p-value 0.01), between 5 and 10% (light red) and above 10% (dark red). The three complementarity determining regions (CDRs) are easily identifiable by HDX-MS, as they incorporate more deuterium than other regions of the protein.

- **BRIL region, as a negative control for our HDX-MS experiments**


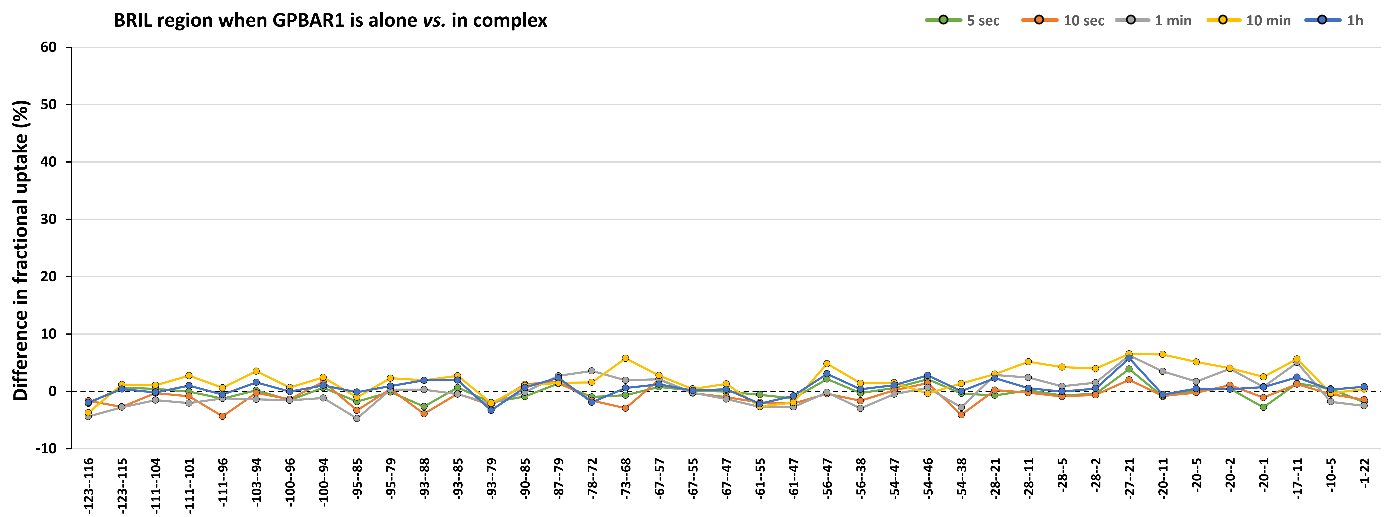
Figure S9: Differences in relative D incorporation BRIL region when GPBAR1 is alone and in complex within GPBAR1/Gs for each peptide identified at different deuteration times. The framed peptides show statistically significant differences in D incorporation (MEMHDX, p-value 0.01).


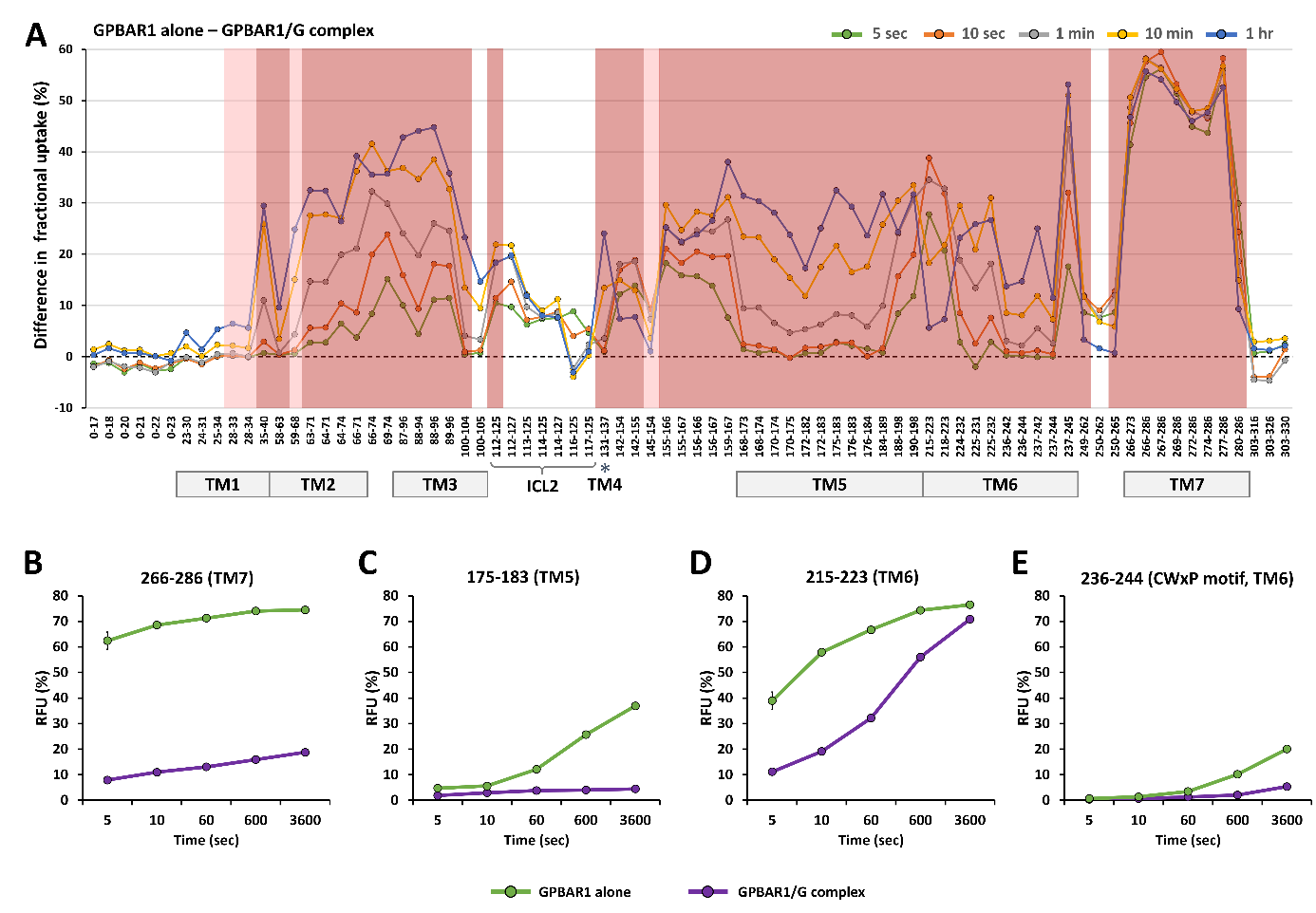


Figure S10: Differential HDX-MS (ΔHDX) of apo GPBAR1 and GPBAR1/G_s_ complex. Differences in relative D uptake between apo-GPBAR1 alone and complexed for each peptide identified at different deuteration times. Boxed peptides show statistically significant differences in D incorporation (MEMHDX, p-value 0.01), between 5 and 10% (light red) and above 10% (dark red).


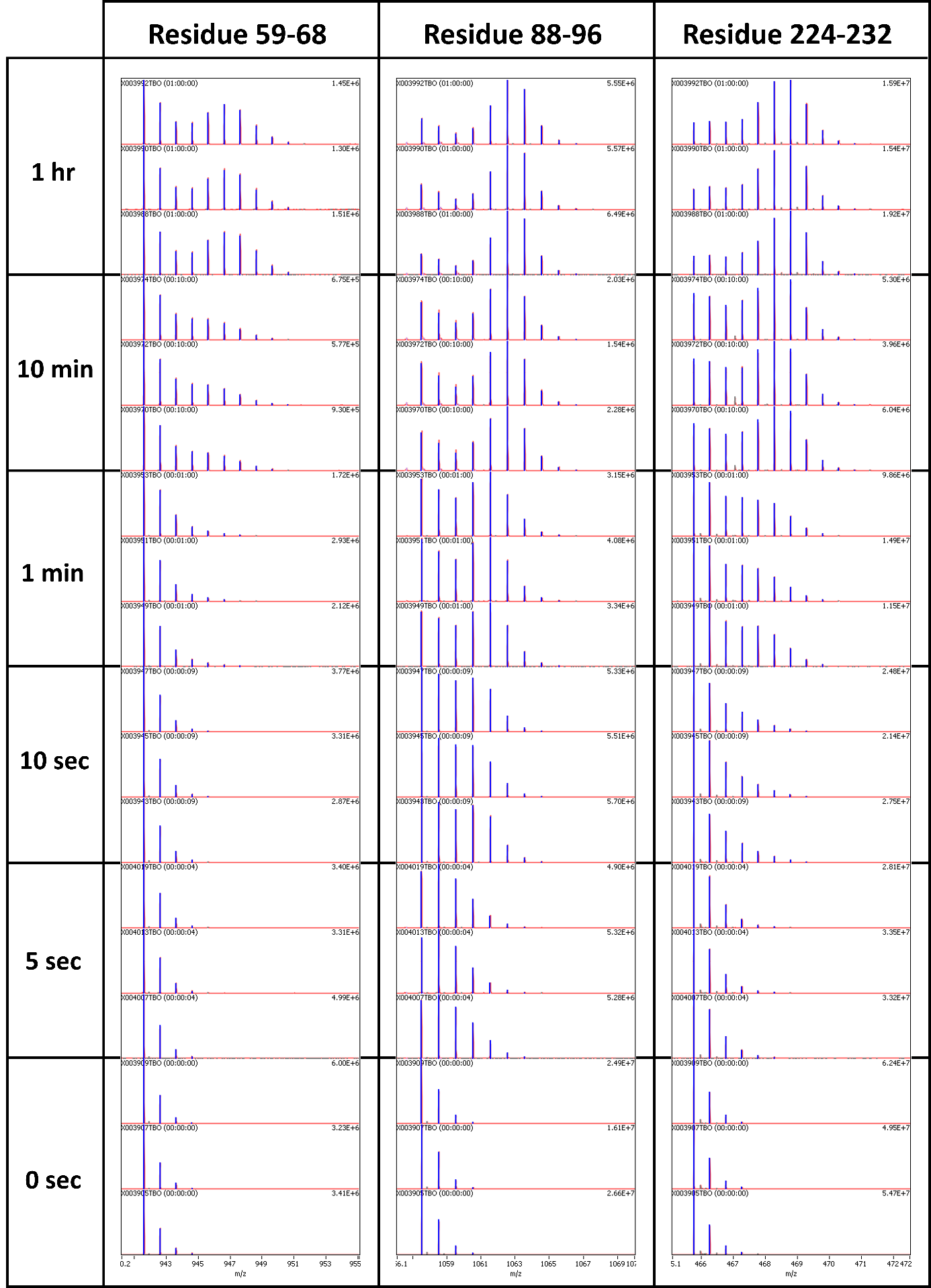


Figure S11: Mass spectra of residues 59-68 (TM2 region), 88-96 (TM3 region) and 224-232 (TM6 region) after digestion and labeling of GPBAR1 alone from 5 seconds to 60 minutes. Each labeling condition has been performed in triplicates. These three peptides display a characteristic bimodal distribution for apo-GPBAR1 state.

- **Back-exchange correction**


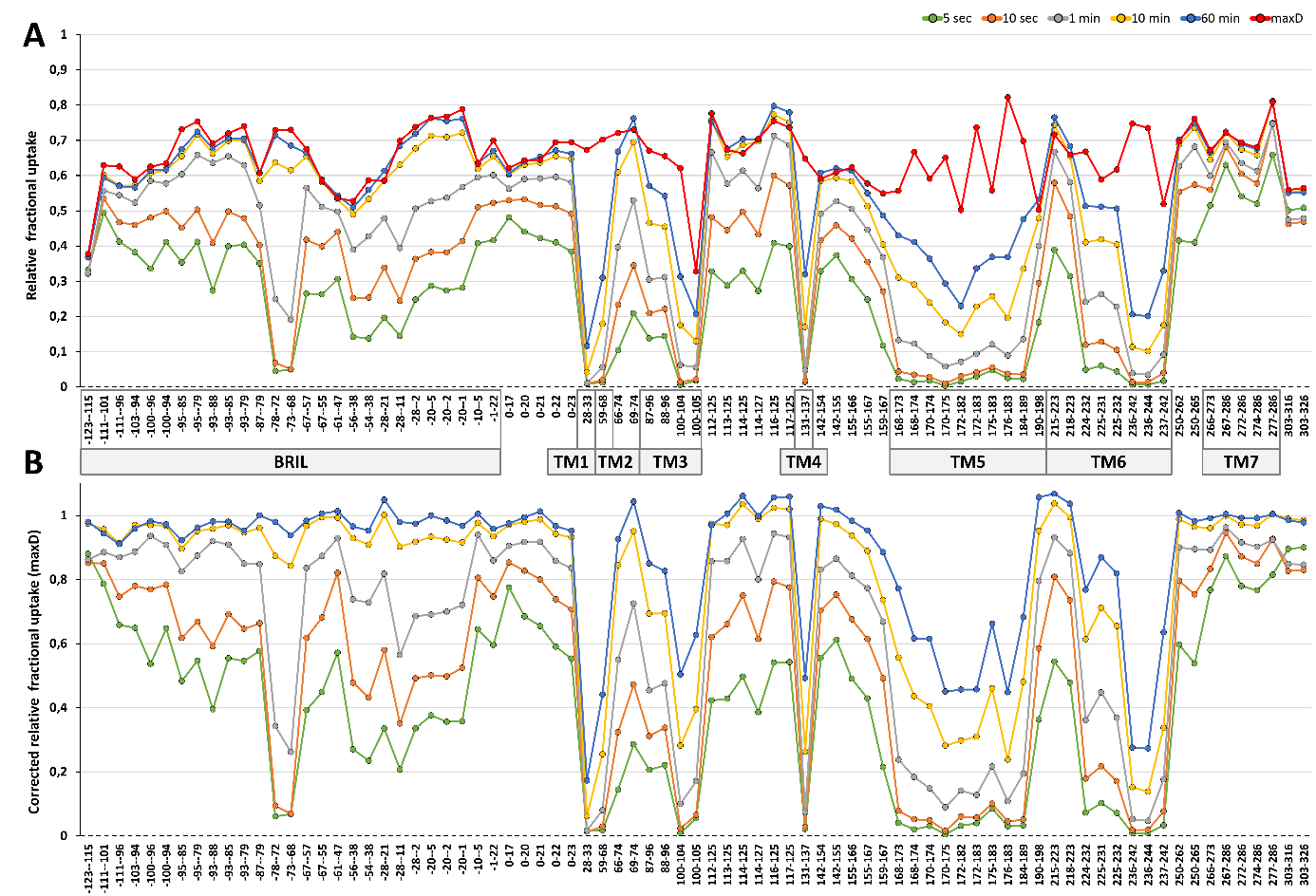


Figure S12: Representation of the relative D incorporation of GPBAR1 alone for each peptide identified at different deuteration times without (A) and with (B) back-exchange correction. The peptides resulting from the digestion of the BRIL protein are numbered from -123 to 0 while those resulting from GPBAR1digestion are numbered from 1 to 326.


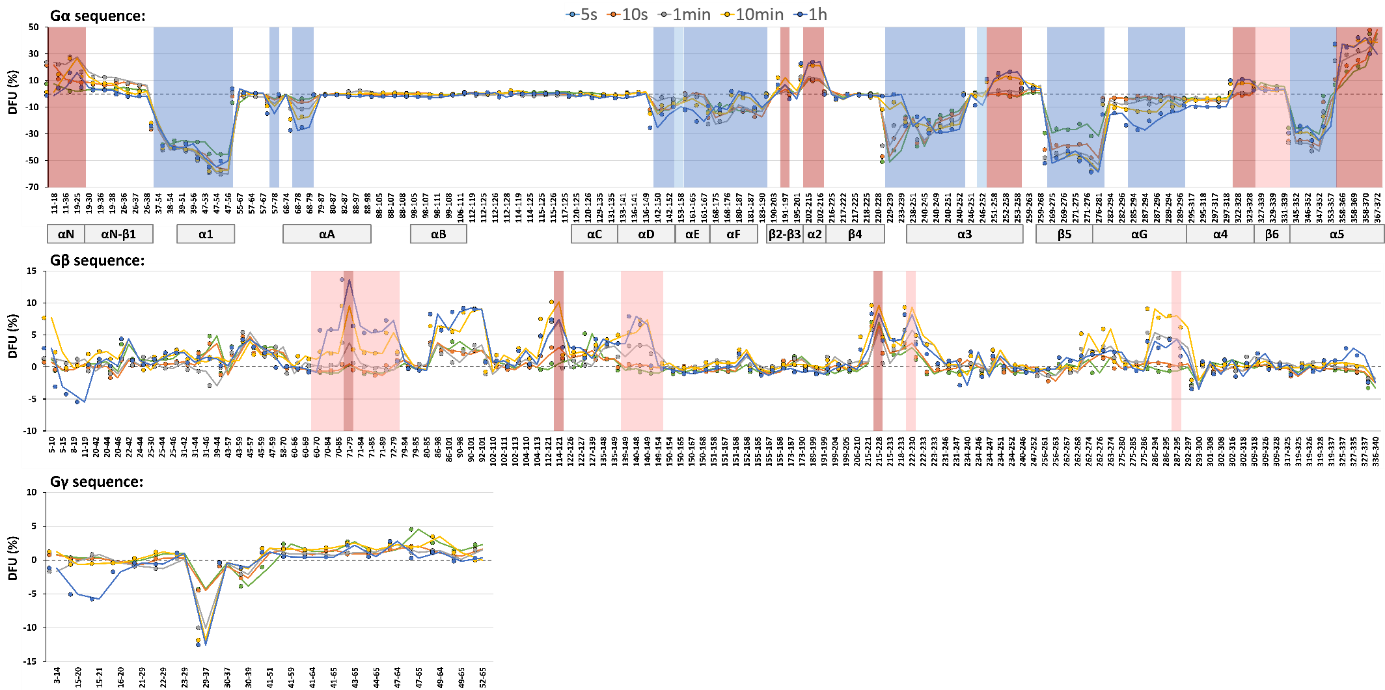


Figure S13: Relative differences in D uptake for (A) Gαs, (B) Gβ, and (C) Gγ when the G protein is alone or in complex with GPBAR1 for each peptide identified at different deuteration times. Boxed peptides show statistically significant differences in D incorporation (MEMHDX, p-value 0.01). Peptides framed in red show a decrease in solvent accessibility after complex formation (protection), while those in blue show an increase (deprotection).

- Statistical analysis and peptide clustering


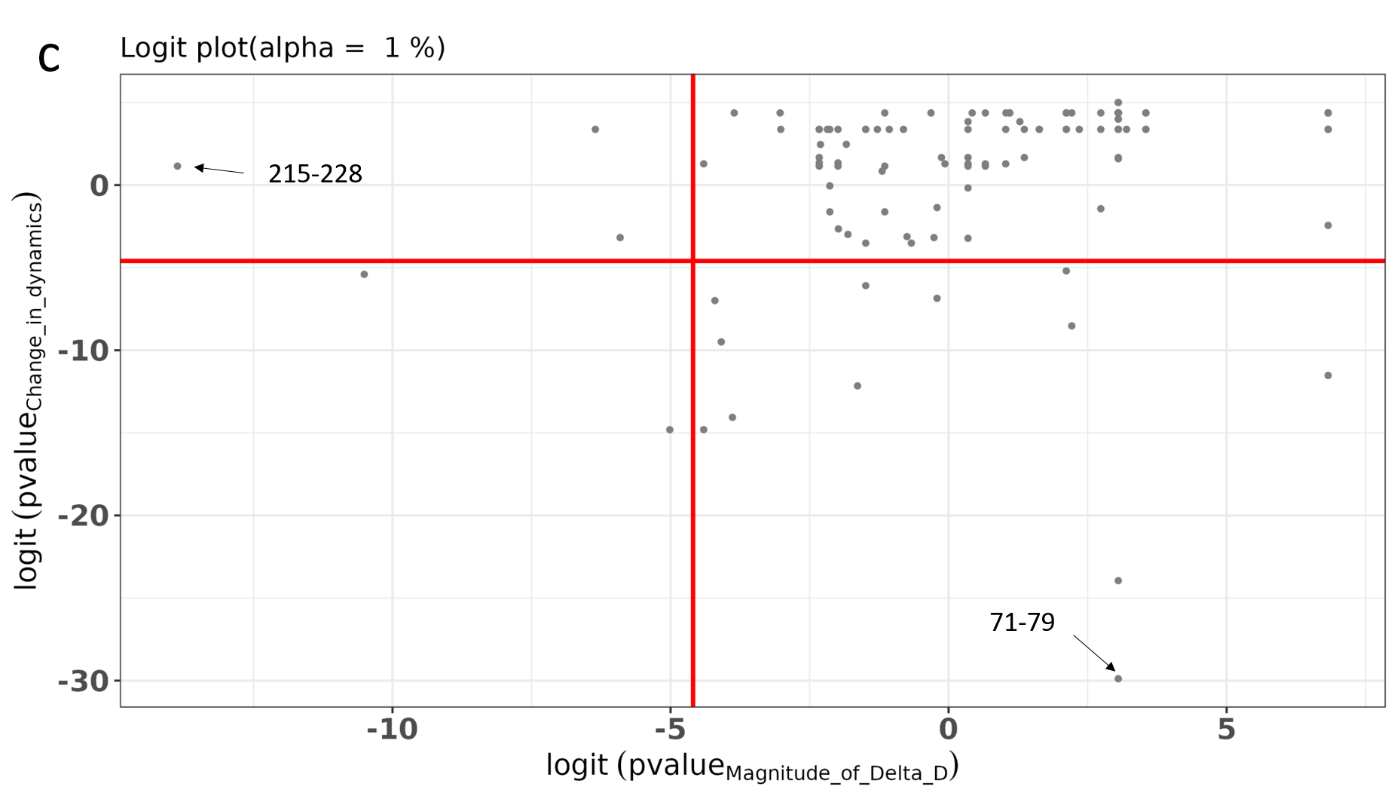


Figure S14: Logit plots generated with MEMHDX for Gβ. Each dot corresponds to one unique peptide. Peptides are classified based on their respective HDX behavior. MEMHDX automatically summarizes and displays the statistical results by means of a ‘Logit’ representation. Two adjusted P-values are calculated by the software and define the position of each peptide in the ‘Logit’ plot. On one hand, the magnitude of ΔHDX-associated P-value defines the magnitude of difference of deuterium uptake between the two states investigated, while on the other hand, the change in dynamics associated P-value describes the timewise evolution of HDX behavior between states. Considering the magnitude of ΔHDX only, the significance increases (i.e. P-value decreases) as the peptide position moves from right to left on the plot, due to the greater magnitude of change observed between states. Similarly, the significance of change in dynamics increases as the peptide position moves from top to bottom. The statistical significance threshold was set to 1%.
